# Impact of thermal processing on polyphenols, carotenoids, glucosinolates, and ascorbic acid in fruit and vegetables and their cardiovascular benefits

**DOI:** 10.1111/1541-4337.13426

**Published:** 2024-08-21

**Authors:** Federica Narra, Eugenia Piragine, Giada Benedetti, Costanza Ceccanti, Marta Florio, Jacopo Spezzini, Fabiola Troisi, Roberto Giovannoni, Alma Martelli, Lucia Guidi

**Affiliations:** ^1^ Department of Agriculture, Food and Environment University of Pisa Pisa Italy; ^2^ Interdepartmental Research Center Nutrafood “Nutraceuticals and Food for Health” University of Pisa Pisa Italy; ^3^ Department of Pharmacy University of Pisa Pisa Italy; ^4^ Department of Biology University of Pisa Pisa Italy

**Keywords:** ascorbic acid, cardiovascular diseases, carotenoids, glucosinolates, polyphenols

## Abstract

Bioactive compounds in fruit and vegetables have a positive impact on human health by reducing oxidative stress, inflammation, and the risk of chronic diseases such as cancer, cardiovascular (CV) diseases, and metabolic disorders. However, some fruit and vegetables must be heated before consumption and thermal processes can modify the amount of nutraceuticals, that is, polyphenols, carotenoids, glucosinolates, and ascorbic acid, that can increase or decrease in relation to different factors such as type of processing, temperature, and time but also the plant part (e.g., flower, leaf, tuber, and root) utilized as food. Another important aspect is related to the bioaccessibility and bioavailability of nutraceuticals. Indeed, the key stage of nutraceutical bioefficiency is oral bioavailability, which involves the release of nutraceuticals from fruit and vegetables in gastrointestinal fluids, the solubilization of nutraceuticals and their interaction with other components of gastrointestinal fluids, the absorption of nutraceuticals by the epithelial layer, and the chemical and biochemical transformations into epithelial cells. Several studies have shown that thermal processing can enhance the absorption of nutraceuticals from fruit and vegetable. Once absorbed, they reach the blood vessels and promote multiple biological effects (e.g., antioxidant, anti‐inflammatory, antihypertensive, vasoprotective, and cardioprotective). In this review, we described the impact of different thermal processes (such as boiling, steaming and superheated steaming, blanching, and microwaving) on the retention/degradation of bioactive compounds and their health‐promoting effects after the intake. We then summarized the impact of heating on the absorption of nutraceuticals and the biological effects promoted by natural compounds in the CV system to provide a comprehensive overview of the potential impact of thermal processing on the CV benefits of fruit and vegetables.

## 1 INTRODUCTION

Fruit and vegetables contain many antioxidant and anti‐inflammatory compounds, making them a positive addition to the human diet. Therefore, high consumption of fruit and vegetables represents part of the practical and likely strategy to prevent many degenerative diseases associated with subclinical oxidative stress and inflammation, such as cancer, metabolic disorders, and cardiovascular diseases (CVDs) (Gunathilake et al., 2018; Moyo et al., 2020).

Nutraceuticals play a crucial role in reducing oxidative stress and inflammation (Loizzo & Tundis, 2019), including direct scavenging of reactive oxygen species (ROS), chelation of metals involved in the Fenton reaction, and activation of the endogenous antioxidant pathways (Simunkova et al., 2021). Among the bioactive compounds present in fruit and vegetables, we focused on phenolic compounds, carotenoids, glucosinolates (GLs), and ascorbic acid to delve into the specifics of antioxidant molecule behavior when fruit and vegetables are subjected to thermal processes.

Polyphenols, including flavonoids, lignans, phenolic acids, and stilbenes (Prabhu et al., 2021), are widely distributed in the plant kingdom, and they have been found in vegetables such as cabbage, onion, broccoli, and spinach and in many other fruit and vegetables (Andrés‐Lacueva et al., 2009).

Carotenoids, including β‐carotene, α‐carotene, γ‐carotene, lycopene, zeaxanthin, violaxanthin, and lutein (Szabo et al., 2018), play a crucial role in protecting cell membranes by scavenging or deactivating ROS (Fiedor & Burda, 2014; Tapiero et al., 2004). They have been found in vegetables and fruit such as carrots, pumpkins, melons, apricots, tomatoes, watermelons, and peppers (Mangels et al., 1993).

GLs are secondary metabolites present in vegetables belonging to the *Brassicaceae, Capparidaceae*, and *Euphorbiaceae* (Campos‐Vega & Oomah, 2013), which have a recognized role in reducing the risk of chronic and degenerative diseases, including cancer, CVDs, and metabolic disorders (Arikawa & Gallaher, 2008; Kapusta‐Duch et al., 2016). Many of the health‐promoting properties attributed to GLs are due to the effects of secondary metabolites derived from myrosinase‐induced hydrolysis of GLs, that is, the isothiocyanates (ITCs; Baenas et al., 2019; Citi et al., 2019; Oloyede et al., 2021).

Finally, a naturally occurring compound in vegetables is ascorbic acid, commonly known as vitamin C when present in its reduced form. Vitamin C is one of the most important exogenous vitamins, that is recognized for its antioxidant and anti‐inflammatory properties (Gegotek & Skrzydlewska, 2022). Ascorbic acid is highly present in pepper, kiwi, cauliflower, spinach, broccoli, and citrus fruit (Vincente et al., 2014).

## 2 THERMAL PROCESSING

Different thermal processes can induce alterations in food components and influence the physiochemical properties of foods, including color, texture, flavor, and nutritional value (López‐Hernández et al., 2022; Wainaina et al., 2021). In the case of fruit and vegetables, composition and concentration of plant secondary metabolites are highly species‐dependent, and the stability of these compounds depends on their chemical structure (Chen et al., 2019). Several vegetables are consumed raw. However, most vegetables to make them edible are frequently consumed in the thermal treated form as part of the human diet. Thermal treatments, such as boiling, steaming, blanching, and microwaving, affect the stability and bioactivity of plant secondary metabolites (Arfaoui, 2021; Rashmi & Negi, 2020). Indeed, it is well known that heat treatments lead to a reduction in the nutritional value of fruit and vegetables, altering the chemical composition, content, and bioavailability of bioactive compounds (Paciulli et al., 2018). These different effects are attributed to various thermal processing conditions, including treatment duration (range between 1 and 60 min), water content, and microwave power (range between 560 and 700 W) (Lee et al., 2018; Yang et al., 2016). Some nutraceutical compounds, such as some carotenoids in bitter melon fruit, are highly susceptible to degradation when exposed to heat (Kim et al., 2023), whereas polyphenols in whole plant of Japanese honeysuckle have shown certain stability when subjected to high temperatures (100°C) but for relatively short durations (Lee et al., 2019).

**FIGURE 1:**
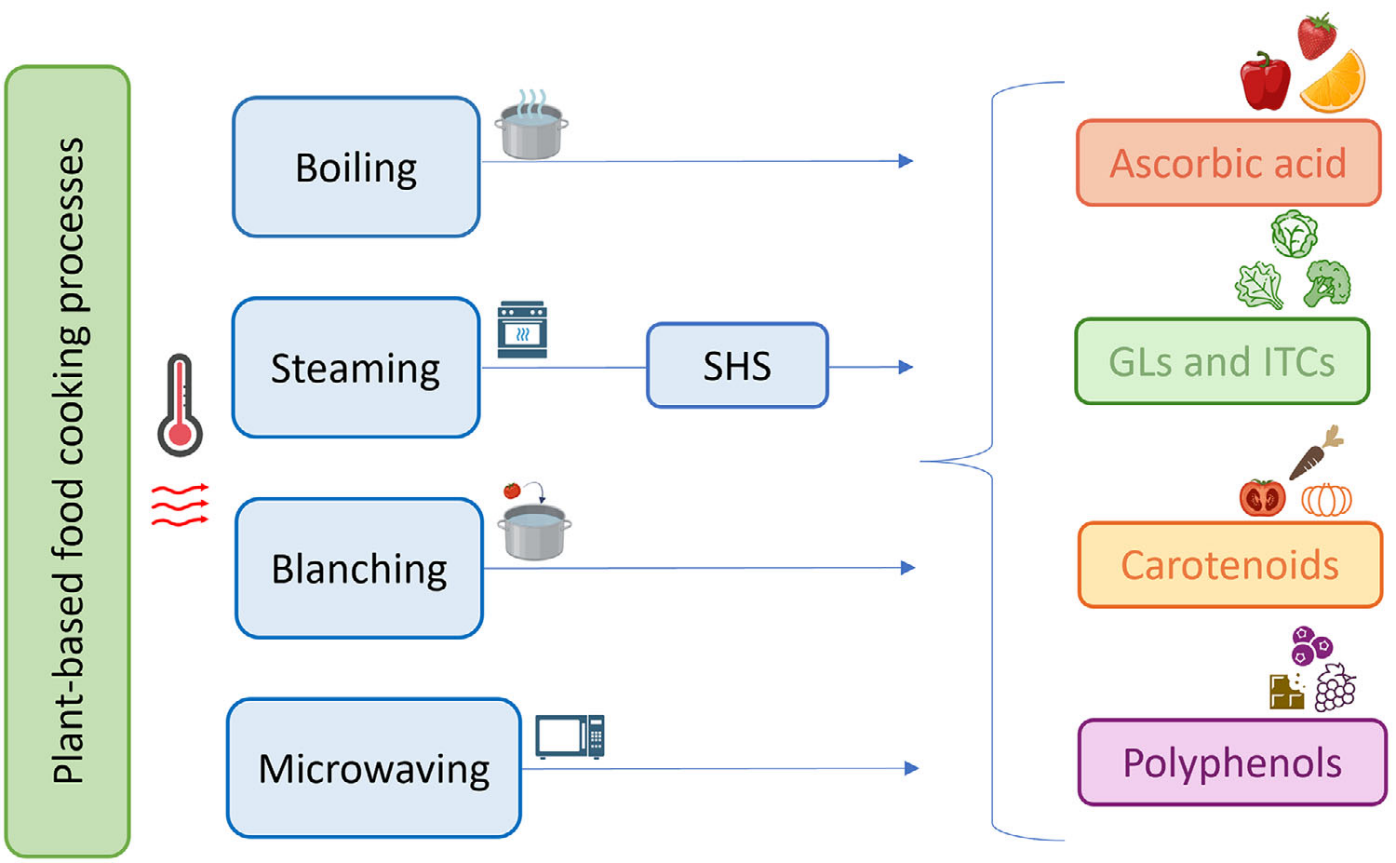
Effects of thermal processing on bioactive compounds present in fruit and vegetables. GLs, glucosinolates; ITCs, isothiocyanates; SHS, superheated steam.

**FIGURE 2:**
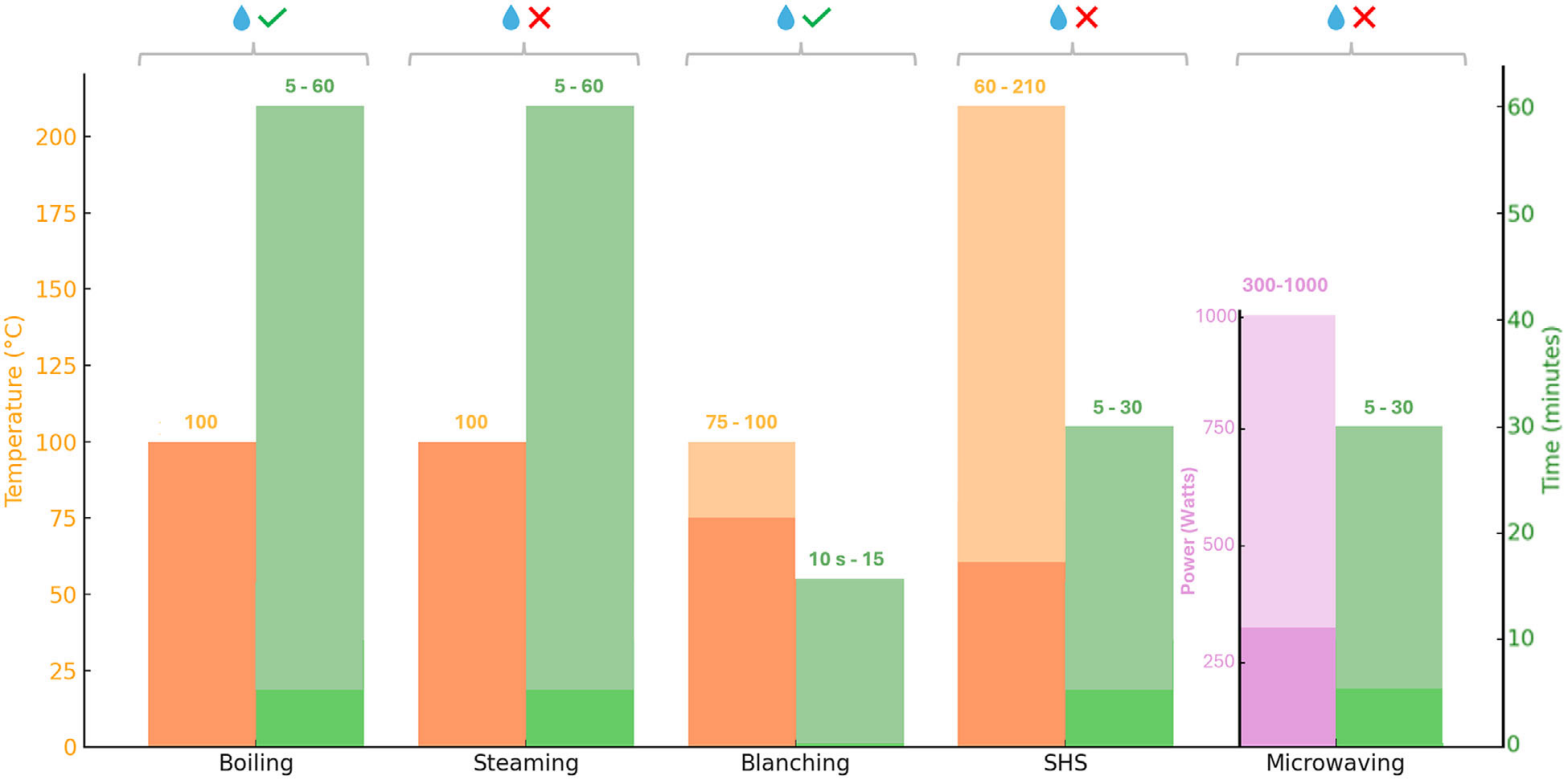
Temperature (orange bars), power (pink bar), time (green bars), and use of water (represented by a drop) in different thermal processes. Color intensity represents the different ranges for each parameter. SHS, superheating steaming.

Many studies investigated how different heat treatments affect the phytochemical composition and antioxidant activity of fruit and vegetables in the human diet. In this review, a survey has been conducted on the effects of thermal processes such as boiling, steaming, and superheated steaming (SHS), blanching, and microwaving on selected classes of bioactive compounds such as polyphenols, carotenoids, GLs, and ITCs, and ascorbic acid (Figure 1). Figure 2 summarizes the thermal characteristics of each process described in the next sections.

### 2.1 Boiling

Boiling is a traditional thermal process involving the immersion of plant or animal‐based food, in heated water until it reaches its boiling point. Table 1 reports a summary of recent works regarding boiling effects on bioactive compounds in fruit and vegetables. The majority of studies reported a decrease of total phenolic content (TPC), total flavonoid content (TFC), and antioxidant activity in different plant species (see Table 1) independently to the boiled edible plant portion and the boiling time (for the references see Table 1). Indeed, phenolic compounds are poorly stabilized and easily degraded by heat, and they are lost in large quantities during heating (Zhang et al., 2023). A recent study showed that boiling tatsoi leaves for 5 min caused a significant decrease in TPC (−27.1%) compared to the raw leaves due to the breakdown and structural changes of phenolic compounds and their leaching into the water (Yang et al., 2019). This hypothesis was endorsed by many other authors (Ceccanti, De Bellis, et al., 2022; Dominguez‐Fernandez et al., 2021; Lidikova et al., 2021; Llorent‐Martínez et al., 2020; Salamatullah, Hayat, et al., 2021; Salamatullah, Özcan, et al., 2021; Zhang et al., 2023). In fact, a 25% reduction in TPC of artichoke heads (private of external leaves) boiled for 15 min, compared to raw samples, was reported (Dominguez‐Fernandez et al., 2021). The authors also observed that the discarded water of the boiling process contained a significant amount of both TPC and TFC, demonstrating the effective leaching of phenolic compounds in the boiling water (Dominguez‐Fernandez et al., 2021). Moreover, boiling can disrupt the interactions between tannin and starch molecules, leading to the degradation of phenolic polymers and a reduction in tannin content, and the destruction of the tannin structure may contribute to the reduction in TPC (Zhang et al., 2023). Another explanation of the decrease of TFC and TPC could be attributed to the formation of early Maillard reactions products with prooxidant properties. Therefore, the depletion of antioxidant compounds in boiled vegetables may be attributed to the consumption of phenolic compounds as reactants in the Maillard reaction (Kaanane & Labuza, 1989). Furthermore, authors agree that boiling produces a loss in both TPC and individual phenolic compounds (Llorent‐Martínez et al., 2020). Indeed, specific losses in cinnamate esters (−78%), sinapic acid (−100%), and coumaric acid derivatives (−54%) were found (Llorent‐Martínez et al., 2020). However, the differences in terms of specific phenolic compounds varied more with plant species or edible plant portion than with the type of thermal process (Rothwell et al., 2015). For this reason, patterns of retention or degradation of flavonoids and other phenolic compounds in the literature are different and numerous.

**TABLE 1:** Effect of boiling on bioactive compounds in vegetables.

Plant species	Cooking conditions	Phytochemical changes	References
Onion (*Allium cepa*)	100°C; 10 min	= TPC ↓Ascorbic acid ↓Antioxidant activity (DPPH, FRAP, and ABTS)	Lidikova et al. (2021)
	100°C; 5, 15, and 30 min	↑TPC (at 15 and 30 min) ↑TFC ↑FRAP (at 30 min) and DPPH (at 5 min)	Kam et al. (2023)
Garlic (*Allium sativum*)	100°C; 10 min	↓TPC ↓Ascorbic acid ↓Antioxidant activity (DPPH, FRAP, and ABTS)	Lidikova et al. (2021)
*Alpinia zerumbet*	100°C; 10, 20, and 30 min	↓TPC (after 30 min when compared with 10 and 20 min) ↓TFC (after 30 min when compared with 10 and 20 min) = DPPH	da Cruz et al. (2023)
Celery (*Apium graveolens*)	100°C; until the softening	↓TPC and DPPH	Salamatullah, Hayat et al. (2021)
	100°C; 10 ± 5 min	↓Ascorbic acid ↑TPC	Zor et al. (2022)
Peanut (*Arachis hypogaea*)	100°C; 10 min	↓TPC ↓TFC ↓DPPH, ABTS, and FRAP	Zhang et al. (2023)
Horseradish (*Armoracia rusticana*)	100°C; 60 min	↓TPC ↑TFC ↓ABTS and DPPH	Karafyllaki et al. (2023)
Beetroot (*Beta vulgaris*)	100°C; 10 ± 5 min	↓Ascorbic acid ↓TPC	Zor et al. (2022)
Borage (*Borago officinalis*)	100°C; 2 min	↓TPC ↓DPPH	Ceccanti, Landi et al. (2022)
Broccolini (*Brassica alboglabra*)	100°C; 3 min	↓TPC (phenolic acids and flavonoids)	Llorent‐Martínez et al. (2020)
	97 ± 2°C; 1, 2, and 3 min	↑TPC ↑ABTS and DPPH	Chin et al. (2022)
Chinese cabbage (*Brassica chinensis*)	100°C; 4, 8, and 12 min	↓TPC ↓TFC = FRAP ↑DPPH	Kam et al. (2023)
	100°C; 10 and 15 min	↓TPC ↑TCC	Chen et al. (2019)
Green mustard (*Brassica juncea*)	100°C; 5 min	↓Ascorbic acid ↑β‐Carotene ↓TPC = TFC ↑DPPH	Rana et al. (2021)
Brussels sprouts (*Brassica oleracea*)	100°C; 8 min	↑GLS ↓ITC	Wieczorek et al. (2023)
	100°C; 15 min (+different concentrations of KCl or NaCl)	↓Caffeic acid ↓*p*‐Coumaric acid	Doniec et al. (2022)
White rose cauliflower (*Brassica oleracea* var. *Botrytis*)	100°C; 8 min (+different concentrations of KCl or NaCl)	↓Protocatechuic acid ↓Caffeic acid ↓Sinapic acid	Doniec et al. (2022)
Broccoli (*Brassica oleracea* var*. Italica*)	100°C; 8 min (+different concentrations of KCl or NaCl)	↓Protocatechuic acid ↓Caffeic acid ↓*p*‐Coumaric acid ↓Ferulic acid ↓Sinapic acid	Doniec et al. (2022)
	100°C; 5 min	↓TPC ↓DPPH	Czarnowska‐Kujawska et al. (2022)
	100°C; 8 min	↑TCC	de Castro et al. (2021)
	100°C, 20 min	↓TPC ↑DPPH ↓Ascorbic acid = GLS	Tabart et al. (2018)
Cabbage (*Brassica oleracea* var. *capitata*)	100°C; 5 min	↓Ascorbic acid ↑β‐Carotene = TPC and TFC ↑DPPH	Rana et al. (2021)
Red cabbage (*Brassica oleracea* var. *capitata rubra*)	100°C; 10, 15, 20, and 25 min	↓TPC ↓TFC ↓DPPH, FRAP, and ABTS	Avila et al. (2023)
	100°C; 5 min	↓TPC and TAC ↓DPPH and FRAP	Tan et al. (2023)
	100°C; 1, 3, 5, 10, 20, and 30 min	↓GLS	Wu et al. (2021)
	100°C, 20 min	↓TPC = DPPH ↓Ascorbic acid ↓GLS	Tabart et al. (2018)
	100°C; 5, 10, 30, 60, 90, or 120 min	↓GLS ↑GLS (until 10 min) ↑Nitriles (until 120 min)	Renz et al. (2024)
Tatsoi (*Brassica rapa* subsp*. narinosa)*	100°C; 5 min	↓TPC ↓GLS ↓Ascorbic acid ↓DPPH	Yang et al. (2019)
Turnip (*Brassica rapa* subsp. *rapa*)	100°C; 10 ± 5 min	↓Ascorbic acid ↓TPC	Zor et al. (2022)
Pepper (*Capsicum annuum*)	100°C; 10 min	↓TPC ↓Ascorbic acid ↓Antioxidant activity (DPPH, FRAP, and ABTS)	Lidikova et al. (2021)
Quinoa (*Chenopodium quinoa*)	100°C; 2 min	= TPC = TFC = PPH ↓ABTS	Zhang et al. (2022)
	100°C; 10, 15, and 20 min	↓TPC (at 15 and 20 min when compared with raw samples) = TFC ↓DPPH, ABTS, FRAP	Sharma et al. (2022)
Spider plant (*Cleome gynandra*)	100°C, 120 min	↑TPC ↑TFC	Moyo et al. (2016)
Pumpkin (*Cucurbita moschata*)	100°C; 10 min	↑TCC	de Castro et al. (2021)
	98°C; 10 min	↑TPC ↑DPPH ↓ABTS	Mashitoa, Manhivi et al. (2021)
Artichoke (*Cynara cardunculus* var. *scolymus*)	100°C; 15 min	↓TPC ↓TFC	Dominguez‐Fernandez et al. (2021)
Cardoon (*Cynara cardunculus* var. *altilis* cv. Lucchese)	100°C; 30 min	↑TPC ↑DPPH ↓Cynaropicrin	Ceccanti, De Bellis et al. (2022)
cv. Plain Blanc Inerme	100°C; 30 min	= TPC ↑DPPH ↓Cynaropicrin	Ceccanti, De Bellis et al. (2022)
Tiger nuts (*Cyperus esculentus*)	98°C; 20, 30, and 40 min	↑TPC (at 20 and 30 min) ↑DPPH (at 40 min)	Djikeng et al. (2022)
Carrot (*Daucus carota*)	100°C; 10 ± 5 min	↓TPC	Zor et al. (2022)
	100°C; 10 min	↓TCC	de Castro et al. (2021)
var. *purple haze*	100°C; 10 min	↑TCC ↓TPC and TAC	Sáez‐Escudero et al. (2023)
Jerusalem artichoke (*Helianthus tuberosus*)	100°C; 10 ± 5 min	↓Ascorbic acid = TPC	Zor et al. (2022)
Sweet potato (*Ipomoea batatas*)	100°C; 2 min	↓TPC	Musilova et al. (2020)
Green bean (*Lablab purpureus*)	100°C; 5 min	↓Ascorbic acid = β‐Carotene ↓TPC and TFC ↑DPPH	Rana et al. (2021)
Mallow (*Malva sylvestris*)	100°C; 2.5 min	↓TPC ↑DPPH	Ceccanti, Landi et al. (2022)
African pumpkin (*Momordica balsamina*)	100°C; 20 min	↑Most of flavonoids and phenolic acids (quercetin‐3‐*O*‐glucoside, dicaffeoylquinic acids, etc.) = TPC = TEAC, ORAC, and FRAP	Moyo et al. (2021)
	98°C; 15 min	↓DPPH and ABTS ↑FRAP ↑β‐Carotene ↓Most of flavonoids	Mashiane et al. (2021)
	98°C; 15 min	↑TCC (lutein and β‐carotene) ↓Zeaxanthin ↑DPPH and ABTS	Mashiane et al. (2022)
Drumstick (*Moringa oleifera*)	100°C; 4, 8, and 12 min	↑TPC ↑TFC (at 8 and 12 min) ↑FRAP and DPPH (at 12 min)	Kam et al. (2023)
	100°C; 2 min	↓β‐Carotene ↓Ascorbic acid	Kachhawa and Chawla (2024)
Watercress (*Nasturtium officinale*)	90°C; 2, 5, and 10 min	↓TFC after 2, 5, and 1 min ↓GLS after 2, 5, and 10 min ↑TCC after 2 and 5 min	Giallourou et al. (2016)
Basil (*Ocimum sanctum*)	100°C; 5, 10, and 15 min	↓TPC (at 15 min when compared with 5 and 10 min) ↓TFC (at 15 min when compared with 5 min) ↑DPPH (at 10 and 15 min when compared with 5 min) ↓FRAP (at 15 min when compared with 5 min)	Salamatullah, Hayat et al. (2021)
Pohpohan (*Pilea trinervia*)	100 and 70°C; 5 and 15 min	↓TPC ↓DPPH	Ardiansyah et al. (2023)
Buck's‐horn plantain (*Plantago coronopus*)	100°C; 8 min	↓TPC ↓DPPH	Ceccanti, Landi et al. (2022)
Rosemary (*Rosmarinus officinalis*)	100°C; 5, 10, and 15 min	↑TPC (at 15 min when compared with 5 min) ↑TFC (at 10 and 15 min when compared with 5 min) ↓DPPH (at 15 min when compared with 10 and 5 min) ↑FRAP (at 15 min when compared with 5 min)	Salamatullah, Hayat et al. (2021)
Marula fruit (*Sclerocarya birrea* subsp*. caffra*)	100°C; 5 min	↓Ascorbic acid ↑TPC ↑ABTS and DPPH ↑TCC	Dorothy et al. (2023)
	100°C; 10 min	↓TPC ↓Ascorbic acid ↓Antioxidant activity (DPPH, FRAP, and ABTS)	Lidikova et al. (2021)
Potato (*Solanum tuberosum*)	100°C; 10 min	= TPC ↓Ascorbic acid ↓DPPH and ABTS = FRAP	Lidikova et al. (2021)
	100°C; 10 ± 5 min	↓Ascorbic acid ↓TPC	Zor et al. (2022)
Spinach (*Spinacea oleracea*)	100°C; 2, 4, 6, 8, and 10 min	↑TCC (between 4 and 8 min) ↑TPC (between 4 and 8 min) ↓TFC ↑TAC ↓DPPH	Mehmood and Zeb (2023)
	100°C; 2 min	↓TPC ↓DPPH	Czarnowska‐Kujawska et al. (2022)
	100°C; 20 min	↓TPC and TFC = TEAC ↑ORAC ↓FRAP	Moyo et al. (2021)
Wakame (*Undaria pinnatifida*)	100°C; 10 min	↓TPC	Jiang et al. (2022)
Faba (*Vicia faba*)	100°C; 1, 5, 10, and 15 min	↓TPC = TFC = DPPH ↓ABTS	Duan et al. (2022)

*Note*: Plant food species are in an alphabetical order, considering the species Latin name.

Abbreviations: ABTS, 2,2′‐azino‐bis(3‐ethylbenzothiazoline‐6‐sulfonic acid) antioxidant activity assay; DPPH, 2,2‐diphenyl‐1‐picrylhydrazyl antioxidant activity assay; FRAP, ferric reducing‐antioxidant power assay; GLS, glucosinolates; ITC, isothiocyanates; ORAC, oxygen radical absorbance capacity assay; TAC, total anthocyanin content; TCC, total carotenoid content; TEAC, trolox equivalent antioxidant assay; TFC, total flavonoid content; TPC, total phenolic content.

At the same time, some authors explored the use of salt (either KCl or NaCl) in boiling water to simulate the real‐life conditions that plant foods undergo before reaching consumers (Doniec et al., 2022). They discovered that adding salt resulted in a significant decrease in caffeic acid levels in different vegetables compared to samples boiled without salt. They also noticed a decrease in ferulic acid levels in boiled vegetables with added salt, except for 1 and 3% NaCl concentrations, which increased hydroxycinnamic acid content, as well as a 3% NaCl concentration, which led to an increase in *p*‐coumaric acid content. Generally, the addition of salt created an osmotic imbalance that caused more significant water loss from vegetables, particularly at higher salt concentrations. This, in turn, resulted in a greater loss of water‐soluble components as polyphenols. However, these variations in the retention of hydroxycinnamic acids were attributed to differences in the physical properties of monovalent cations (Na^+^ and K^+^) in these salts (Doniec et al., 2022). Indeed, smaller ions such as potassium are more hydrated than larger ions such as sodium. This represents an important factor for the movement of ions during thermal processing in water and for the diffusion through channels in the cell plasma membrane.

Several authors reported a retention or an increase in terms of values of TPC, TFC, and antioxidant activity (Ceccanti, Landi, et al., 2022; Chin et al., 2022; Djikeng et al., 2022; Dorothy et al., 2023; Kam et al., 2023; Mashiane et al., 2022; Mashitoa, Manhivi, et al., 2021; Moyo et al., 2016, 2021; Rana et al., 2021; Salamatullah, Hayat, et al., 2021; Zhang et al., 2022; Zor et al., 2022). For example, boiling *Cleome gynandra* leaves for 120 min improved TPC and TFC (Moyo et al., 2016). These results can be attributed to the compartmentalization of phenolic compounds in cell vacuole and the consequent more easily extraction of phenolic compounds thanks to the high temperature utilized during boiling process (Chen et al., 2019; Holland et al., 2020) by disrupting the cell wall structure, releasing bound phenolics, and increasing leaf permeability to solutes and solvents (Ceccanti, Landi, et al., 2022; Garcìa et al., 2021; Salamatullah, Hayat, et al., 2021). These mechanisms are affected by the duration of heat treatment. Some authors, analyzing boiled rosemary leaves after 5, 10, and 15 min of boiling process, observed an increase in TFC after 10 min of process and an increase in TPC and antioxidant activity using ferric reducing‐antioxidant power (FRAP) assay after 15 min of process, due to the heat during boiling which may break the cell wall of the plant material causing the release of the phenolic compounds in the solvent (Salamatullah, Hayat, et al., 2021). At the same time, polyphenols are often esterified with cell wall carbohydrates, and the release of phenolic compounds from the cell walls during boiling can lead to an increase in TPC and TFC (Wu et al., 2018). Moreover, the heat treatment as a boiling process could inactivate polyphenol oxidases (PPO), preventing oxidation and polymerization of polyphenols, and inducing the same results (Yamaguchi et al., 2003).

On the other hand, a significant loss of total GLs content (−59%) in tatsoi leaves boiled for 5 min, when compared with raw samples, was observed (Yang et al., 2019). Recently, these results were confirmed by Renz et al. (2024), who found an increment of GLs breakdown products such as ITCs but also nitriles in boiled red cabbage. However, an increment in GLs was found in boiled Brussels sprouts by other authors who suggested a similar hypothesis already speculated for phenolic compounds about the leaching of antioxidant compounds in boiling water (Ciska et al., 2015; Wieczorek et al., 2023). Moreover, an increase in GLs content in Brussels sprouts boiled for 8 min (Wieczorek et al., 2023) and a retention of GLs in broccoli boiled for 20 min (Tabart et al., 2018) were observed. These results have led to the hypothesis that the higher GLs content in boiled plant samples than in raw ones resulted from the degradation of tissue after the heat treatment. This is because GLs might be partially bonded to the cell walls and be released only after tissue structure disintegration. As a result, the extraction performance from processed vegetables can be more effective than from raw ones, retaining the GLs content (Ciska et al., 2015; Tabart et al., 2018; Wieczorek et al., 2023). On the contrary, ITCs content was lower than that observed in the raw ones, likely due to the inhibition of myrosinase activity due to the high temperature of the boiling process (Wieczorek et al., 2023).

A decrease of ascorbic acid was observed after the boiling process, regardless of the plant species and the duration of the process (Dorothy et al., 2023; Lidikova et al., 2021; Rana et al., 2021; Tabart et al., 2018; Yang et al., 2019; Zor et al., 2022). Indeed, it is well known that this molecule is very susceptible to high‐temperature degradation such as in boiling process (Laing et al., 1978). For example, a significant reduction by 30% of antioxidant activity in correlation with a great loss of ascorbic acid by 32.6%, compared to raw fresh‐cut leaves, was observed (Yang et al., 2019). A decrease in ascorbic acid content was reported in different plant species such as onion, garlic, pepper, tomato, and potato boiled for 10 min (Lidikova et al., 2021).

A different and clear trend was observed in terms of total carotenoid content (TCC) increasing after the boiling process (Chen et al., 2019; De Castro et al., 2021; Giallourou et al., 2016; Mashiane et al., 2022; Mehmood & Zeb, 2023; Rana et al., 2021; Sáez‐Escudero et al., 2023). An increased TCC in three different cultivars of Chinese cabbage boiled for both 10 and 15 min has been shown (Chen et al., 2019). More specifically, a higher β‐carotene content in green mustard leaves boiled for 5 min and an increased lutein and β‐carotene levels in African pumpkin leaves boiled for 15 min (Mashiane et al., 2022), compared with raw samples, were observed (Rana et al., 2021). Carotenoids are lipophilic compounds and, since the boiling process, in most cases, takes place within an aqueous matrix, these compounds cannot be leached but rather can concentrate inside the plant foods due to water loss from the samples. At the same time, the low susceptibility of some carotenoids such as lycopene and β‐carotene to high temperatures and the transition from *trans* isomers to more stable *cis* isomers are already known in the literature (Honda et al., 2015; Shi et al., 2003).

### 2.2 Steaming

In the steaming process, fruit and vegetables are heated by exposure to steam generated through the heating of water beneath the food, thus avoiding direct contact between water and the food. The transfer of heat in this method is facilitated by steam, which effectively cooks the food. Table 2 provides a summary of the most recent literature regarding the effects of steaming on phytochemicals in horticultural products.

**TABLE 2:** Effect of steaming on bioactive compounds in vegetables.

Plant species	Cooking conditions	Phytochemical changes	References
Onion (*Allium cepa*)	100°C; 10 min	= Ascorbic acid = TPC = DPPH, ABTS, and FRAP	Lidikova et al. (2021)
Garlic (*Allium sativum*)	100°C; 10 min	= Ascorbic acid ↓TPC ↓DPPH and ABTS = FRAP	Lidikova et al. (2021)
*Alpinia zerumbet*	100°C; 10, 20, and 30 min	↓TPC (after 30 min when compared with 20 min and with boiling samples) ↑TFC (after 20 and 30 min when compared with 10 min and with boiling samples) = DPPH	da Cruz et al. (2023)
Celery (*Apium graveolens*) (celery)	About 100°C, 10 ± 5 min	↑TPC	Zor et al. (2022)
Peanut (*Arachis hypogaea*)	100°C; 10 min	↓TPC ↓TFC ↓DPPH, ABTS, and FRAP	Zhang et al. (2023)
Beetroot (*Beta vulgaris* subsp. v*ulgaris*)	About 100°C, 10 ± 5 min	↓TPC ↓Ascorbic acid	Zor et al. (2022)
Broccolini (*Brassica alboglabra*)	100°C; 5 min	↓TPC (phenolic acids and flavonoids) ↓ABTS and DPPH	Llorent‐Martìnez et al. (2020)
	97 ± 2°C; 2, 3, and 4 min	↑TPC ↑DPPH and ABTS	Chin et al. (2022)
Chinese cabbage (*Brassica chinensis*)	100°C; 5, 10, and 15 min (hydromethanolic extract)	↓TPC ↓TFC ↓FRAP = DPPH	Kam et al. (2023)
	100°C; 10 and 15 min	= TPC = TFC ↑TCC (lutein, zeaxanthin, α‐carotene, and β‐carotene)	Chen et al. (2019)
Green mustard (*Brassica juncea*)	100°C; 7.5 min	↓Ascorbic acid ↑β‐Carotene = TFC ↑TPC ↓DPPH	Rana et al. (2021)
Brussel sprouts (*Brassica oleracea*)	100°C; 10 min	↑GLS ↓ITC = Nitriles	Wieczorek et al. (2023)
	100°C; 7 min	↑GLS	Florkiewicz et al. (2017)
Cauliflower (*Brassica oleracea* var. *botrytis*)	100°C; 7 min	↑GLS	Florkiewicz et al. (2017)
Cabbage (*Brassica oleracea* var. *capitata*)	100°C; 7.5 min	↓Ascorbic acid ↑β‐Carotene = TFC ↑TPC ↑DPPH	Rana et al. (2021)
Red cabbage (*Brassica oleracea* var*. capitata* f. *rubra*)	100°C; 5 min	↓TAC and TPC ↓DPPH and FRAP	Tan et al. (2023)
	100°C; 15 min	↑TPC = DPPH = Ascorbic acid ↑GLS	Tabart et al. (2018)
	100°C; 10, 15, 20, and 25 min	↓TPC and TFC ↓DPPH, ABTS, and FRAP	Avila et al. (2023)
	100°C; 3 min	↓GLS	Wu et al. (2021)
Broccoli (*Brassica oleracea* var. *italica*)	100°C; 10 min	↑TCC	de Castro et al. (2021)
	100°C; 10 min	↓TPC = DPPH	Czarnowska‐Kujawska et al. (2022)
	100°C; 7 min	↑GLS	Florkiewicz et al. (2017)
	100°C; 15 min	= GLS = ITC	Baenas et al. (2019)
	100°C; 15 min	= TPC ↑DPPH ↓Ascorbic acid = GLS	Tabart et al. (2018)
Kale (*Brassica oleracea* var. *sabellica*)	100°C; 15 min	= GLS = ITC	Baenas et al. (2019)
Tatsoi (*Brassica rapa* subsp. *narinosa*)	100°C; 5 min	= TPC ↓Ascorbic acid ↓GLS	Yang et al. (2019)
Turnip (*Brassica rapa* subsp. *rapa*)	100°C, 10 ± 5 min	↓TPC ↓Ascorbic acid	Zor et al. (2022)
Pepper (*Capsicum annuum*)	100°C; 10 min	↓Ascorbic acid = TPC = DPPH, ABTS, and FRAP	Lidikova et al. (2021)
Ranawara (*Cassia auriculata*)	100°C, 5 min	↑TPC ↑TFC ↑DPPH ↓TCC	Gunathilake et al. (2018)
Gotu kola (*Centella asiatica*)	100°C, 5 min	↑TPC ↑TFC ↑DPPH ↑TCC	Gunathilake et al. (2018)
Chicory (*Cichorium intybus*)	100°C; 10 min	=β‐Carotene and lutein	Fratianni et al. (2021)
Pumpkin (*Cucurbita moschata*)	98°C, 5 min	↑Total phenolic acids ↓ABTS = DPPH	Mashitoa, Manhivi et al. (2021)
	100°C; 12 min	↑TCC	de Castro et al. (2021)
Tiger nuts (*Cyperus esculentus*)	98°C; 20, 30, and 40 min	↑TPC	Djikeng et al. (2022)
Carrot (*Daucus carota*)	100°C; 10 min	=TCC	de Castro et al. (2021)
	100°C, 10 ± 5 min	↓TPC	Zor et al. (2022)
Black carrot (*Daucus carota* ssp. s*ativus* var*. atrorubens*)	100°C, 10 ± 5 min	↓TPC	Zor et al. (2022)
	100°C; 10 min	↓TCC = TPC ↑TAC	Sáez‐Escudero et al. (2023)
Kuringan‐Ceylon cow tree (*Gymnema lactiferum*)	100°C, 5 min	↓TPC = TFC = DPPH ↓TCC	Gunathilake et al. (2018)
Sweet potato (*Ipomoea batatas*)	100°C, 10 ± 5 min	↑TPC ↓Ascorbic acid	Zor et al. (2022)
Green bean (*Lablab purpureus*)	100°C; 7.5 min	↓Ascorbic acid = β‐Carotene = TFC ↑TPC ↑DPPH	Rana et al. (2021)
African pumpkin (*Momordica balsamina*)	98°C; 15 min	↑TCC (β‐carotene and lutein) = Zeaxanthin ↑DPPH and ABTS	Mashiane et al. (2022)
Drumstick (*Moringa oleifera*)	100°C; 5, 10, and 15 min (hydromethanolic extract)	= TPC (after 10 min) and ↑TPC after 15 min ↑TFC ↑FRAP (after 5 and 10 min) and ↓FRAP (after 15 min) = DPPH	Kam et al. (2023)
Mella (*Olax zeylanica*)	100°C, 5 min	↓TPC ↑TFC ↓DPPH ↓TCC	Gunathilake et al. (2018)
Passion fruit (*Passiflora edulis*)	100°C, 5 min	↓TPC ↓TFC ↓DPPH ↓TCC	Gunathilake et al. (2018)
Pohpohan (*Pilea trinervia*)	100°C; 5 and 15 min	↓TPC ↓DPPH ↑Ferulic and caffeic acids	Ardiansyah et al. (2023)
Cuban oregano (*Plectranthus amboinicus*)	100°C, 10 min	↑TPC ↑TFC ↑DPPH	Bhave and Dasgupta (2018)
Marula fruit (*Sclerocarya birrea* subsp. c*affra*)	100°C; 5 min	↓Ascorbic acid ↑TPC ↑ABTS and DPPH ↑TCC	Dorothy et al. (2023)
Kathurumurunga (*Sesbania grandiflora*)	100°C, 5 min	↓TPC = TFC ↓DPPH ↓TCC	Gunathilake et al. (2018)
Tomato (*Solanum lycopersicum*)	100°C; 10 min	↓Ascorbic acid = TPC = DPPH, ABTS, and FRAP	Lidikova et al. (2021)
Potato (*Solanum tuberosum*)	100°C; 10 min	↓Ascorbic acid = TPC = DPPH ↓FRAP and ABTS	Lidikova et al. (2021)
	100°C, 10 ± 5 min	↓TPC ↓Ascorbic acid	Zor et al. (2022)
*Sonchus asper*	100°C; 10 min	↓β‐Carotene and lutein	Fratianni et al. (2021)
*Sonchus oleraceus*	100°C; 10 min	=β‐Carotene and lutein	Fratianni et al. (2021)
Spinach (*Spinacea oleracea*)	3 min	↓TPC = DPPH	Czarnowska‐Kujawska et al. (2022)
	100°C; 10 min	= β‐Carotene ↓Lutein	Fratianni et al. (2021)
Faba (*Vicia faba*)	100°C; 15, 30, 45, and 60 min	= Ascorbic acid ↓TPC and TFC ↓DPPH and ABTS	Duan et al. (2022)

*Note*: Plant food species are in an alphabetical order, considering the species Latin name.

Abbreviations: ABTS, 2,2′‐azino‐bis(3‐ethylbenzothiazoline‐6‐sulfonic acid) antioxidant activity assay; DPPH, 2,2‐diphenyl‐1‐picrylhydrazyl antioxidant activity assay; FRAP, ferric reducing‐antioxidant power assay; GLS, glucosinolates; ITC, isothiocyanates; TAC, total anthocyanin content; TCC, total carotenoid content; TFC, total flavonoid content; TPC, total phenolic content.

In general, the steaming process leads to the depletion of TPC, TFC, and antioxidant activity in fruit and vegetables, especially using longer exposition of fruit and vegetables to the high temperature (100°C) which can induce the same structural changes in phenolic compounds already described for the boiling process, reducing the quantification of phenolic compounds (Ardiansyah et al., 2023; Avila et al., 2023; Czarnowska‐Kujawska et al., 2022; da Cruz et al., 2023; Duan et al., 2022; Gunathilake et al., 2018; Kam et al., 2023; Lidikova et al., 2021; Llorent‐Martínez et al., 2020; Tan et al., 2023; Zhang et al., 2023; Zor et al., 2022). On the other hand, steaming is a thermal process that tends to better retain the nutritional and nutraceutical water‐soluble compounds present in fruit and vegetables compared to boiling. For example, in a comparative analysis between the steaming and boiling processes, some authors investigated the effect of these two thermal processes on three Chinese cabbage cultivars, employing two different times (10 and 15 min) on phenolic acids and flavonoids (Chen et al., 2019). Differently from the boiling process (see Section 2.1), after the steaming, the concentration of phenolic acids and flavonoids remained unchanged when compared to the raw samples (Chen et al., 2019). A similar comparison between boiling and steaming was performed on tatsoi leaves (Yang et al., 2019), confirming the retention of TPC in tatsoi subjected to steaming process at 100°C for 5 min. Moreover, a similar trend was observed in terms of TPC and antioxidant activity evaluated by different assays in onion, potato, pepper, and tomato (Lidikova et al., 2021). In addition, several authors observed an increase in TPC, TFC, and antioxidant activity in steamed vegetables. For example, an increase in TPC, TFC, and antioxidant activity was measured in steamed Cuban oregano leaves than in raw ones (Bhave & Dasgupta, 2018). Recently, an increase in TPC and antioxidant activity assayed by 2,2′‐azino‐bis(3‐ethylbenzothiazoline‐6‐sulfonic acid) (ABTS) and 2,2‐diphenyl‐1‐picrylhydrazyl (DPPH) methods was observed in marula fruit juice treated with water steam for 5 min (Dorothy et al., 2023). Other authors found similar results in steamed potato, celery, red cabbage, drumstick leaves, broccolini, and tiger nuts (Chin et al., 2022; Kam et al., 2023; Tabart et al., 2018; Zor et al., 2022). All these examples confirmed the avoidance of leaching of phenolic compounds and other hydrophilic antioxidants in the water involved in the steaming process but also a major extraction efficiency and rupture of cell wall‐phenol bounds during the steaming process as well as the inactivation of PPO by heat water steam. Moreover, the possibility of producing antioxidants during the Maillard reaction and the release of compounds that can neutralize ROS because of the breakdown of plant material's cell walls, increasing the antioxidant activity, was recently suggested (da Cruz et al., 2023).

To date, few studies are present in the literature about the destiny of GLs and ITCs in steamed *Brassicaceae* vegetables, leading to contrasting results partially due to different heating times (Baenas et al., 2019; Florkiewicz et al., 2017; Tabart et al., 2018; Wu et al., 2021; Yang et al., 2019). An increase in GLs in steamed cauliflower, Brussel sprouts, and broccoli for 7 min was observed (Florkiewicz et al., 2017). Similar results were noted in red cabbage leaves steamed for 15 min (Tabart et al., 2018), whereas a retention of GLs was also observed in broccoli (Baenas et al., 2019; Tabart et al., 2018). Conversely, a decrease in GLs content in tatsoi (Yang et al., 2019) and red cabbage (Wu et al., 2021) leaves was reported. These results suggested an increase of GLs content in steamed plant inflorescences (cauliflower, Brussel sprouts, and broccoli) and a decrease in steamed leaves (tatsoi). An exception is represented by red cabbage in which the duration of steaming determined differences in GLs content, that is, a decrease after 3 min (Wu et al., 2021) and an increase after 15 min of treatment (Tabart et al., 2018). The decrease of GLs content can suggest a hydrolyzation of them in ITCs due to the mechanical injury suffered by the plant tissue, such as chewing or cutting, which allows the myrosinase contact with its substrate. Indeed, myrosinase is in the cytosol of specialist myrosin cells scattered throughout the plant tissue and may also be in the cytosol of other cells (Mithen et al., 2000). Therefore, this enzyme can be strictly closed to GLs once the cell is subjected to heat treatment, cutting, and chewing by consumers (Sarvan et al., 2014). The increase of GLs content found in *Brassicaceae* inflorescences and in red cabbage may be a result of the inactivation of myrosinase activity caused by longer steaming times than those used to cook the leaves (7 or 15 min vs. 3 or 5 min).

As observed in the boiling thermal process, ascorbic acid is very susceptible to high temperatures, and a decrease in the content of this molecule was observed in different steamed plant species (Dorothy et al., 2023; Lidikova et al., 2021; Rana et al., 2021; Tabart et al., 2018; Yang et al., 2019; Zor et al., 2022). Despite these unpromising results, retention of ascorbic acid was reported in some vegetables such as faba leaf, onion and garlic bulb, and red cabbage leaves (Duan et al., 2022; Lidikova et al., 2021; Tabart et al., 2018). These findings suggested that lack of liquid water does not allow the leaching of ascorbic acid, and, at the same time, the steam permits the partial retention of this molecule, likely due to the breakdown of the bond between ascorbic acid and cell walls.

Regarding hydrophobic antioxidant molecules, contrasting results are reported in studies assessing steaming effects on the nutraceutical value of vegetables. However, most of the studies reported a retention or an increase in TCC in several plant foods (Chen et al., 2019; De Castro et al., 2021; Mashiane et al., 2022; Rana et al., 2021). These results agree with those found in boiled vegetables, confirming the low susceptibility of some carotenoids such as lycopene and β‐carotene to degradation at high temperatures.

#### 2.2.1 Superheated steaming (SHS)

SHS is a heat treatment that uses water vapor at a temperature higher than its boiling point (with a maximum of 210°C) at an absolute pressure using a treatment time range from 1 to 30 min. It is considered innovative because, in comparison to conventional thermal processes, SHS can generate a low‐oxygen environment (<0.15%; Ceccanti et al., 2021; Karimi, 2010) able to prevent the oxidation of lipids and the formation of harmful compounds (Fang et al., 2023). In addition, SHS can facilitate the development of desired product quality by reducing nutrient loss and improving the physiochemical properties of food (Fang et al., 2023). Moreover, SHS has proven effective in reducing the presence of microorganisms thanks to the thermal inactivation of various vegetative and spore‐forming bacteria and fungi on the surface of foodstuffs (Rana et al., 2022). Table 3 summarizes the results of studies assessing the impact of the SHS thermal process on fruit and vegetables.

**TABLE 3:** Effect of superheated steaming on bioactive compounds in vegetables.

Plant species	Cooking conditions	Phytochemical changes	References
Kejirak fruit (*Baccaurea pubera*)	170°C; 15 min	↑TPC ↓TFC ↑DPPH ↓FRAP ↓Lycopene	Shaharuddin et al. (2021)
Cabbage (*Brassica oleracea*)	70 and 80°C until reaching dry weight	= GLS ↓Sulforaphane = TPC	Chaisamlitpol et al. (2014)
White cabbage (*Brassica oleracea* var. *capitata*)	60°C; 10 min	↑Sulforaphane	Lekcharoenkul et al. (2014)
Camellia seed oil (*Camellia oleifera*)	120, 150, 180, and 210°C; 5, 10, 15, 20, and 30 min	↑Free phenolics at 210°C after 5, 10, 20, and 30 min and at 180°C after 10, 15, and 30 min ↓Free phenolics at 120 and 150°C after all times, at 180°C after 5 and 20 min and at 210°C after 15 min ↓Conjugated phenolics (except at 120°C after 10 min) ↓Insoluble‐bound phenolics ↓TPC ↑DPPH, ABTS (except for insoluble‐bound phenolics fraction), and FRAP (except for insoluble‐bound phenolic and conjugated phenolic fraction	Wang et al. (2023)
Artichoke (*Cynara cardunculus* var. *scolymus*)	100°C; 6 min	↑Ascorbic acid ↑DPPH ↑TCC	Ceccanti et al. (2021)
Black cumin (*Nigella sativa*)	180°C; 10, 20, and 30 min	↑TPC and TFC (after 30 min) ↑DPPH (after 30 min)	Liang et al. (2018)
Perilla seed oil (*Perilla frutescens*)	150°C; 1, 3, 5, 10, and 20 min	↑TPC ↑DPPH	Lee et al. (2021)
Mango (*Safeda* var.)	60, 70, and 80°C; 30 min (in comparison to vacuum and hot air drying)	↑Ascorbic acid ↑β‐Carotene ↑TPC (at 70 and 80°C) and DPPH	Sehrawat et al. (2018)
Tomato (*Solanum lycopersicum*)	100°C, 4.5 min	= Ascorbic acid ↓DPPH ↓Lycopene	Ceccanti et al. (2021)
Eggplant (*Solanum melongena*)	170°C; 7 min	↑DPPH	Uthumporn et al. (2016)
Spinach (*Spinacia oleracea*)	100°C; 4 min	↑Ascorbic acid ↑DPPH ↑Lutein	Ceccanti et al. (2021)

*Note*: Plant food species are in an alphabetical order, considering the species Latin name.

Abbreviations: ABTS, 2,2′‐azino‐bis(3‐ethylbenzothiazoline‐6‐sulfonic acid) antioxidant activity assay; DPPH, 2,2‐diphenyl‐1‐picrylhydrazyl antioxidant activity assay; FRAP, ferric reducing‐antioxidant power assay; GLS, glucosinolates; TAC, total anthocyanin content; TCC, total carotenoid content; TFC, total flavonoid content; TPC, total phenolic content.

In general, the TPC, TFC, and antioxidant activity were enhanced by the SHS thermal processes (Ceccanti et al., 2021; Lee et al., 2021; Liang et al., 2018; Sehrawat et al., 2018; Shaharuddin et al., 2021). For instance, the SHS thermal process (170°C for 7 min) on two eggplant varieties significantly increased the antioxidant activity assayed by DPPH (+90.2%) (Uthumporn et al., 2016). The same behavior was observed in terms of antioxidant activity in spinach leaves and artichoke inflorescences heated for 4 and 6 min, respectively, showing the independence of the heated plant portion and the time (Ceccanti et al., 2021). SHS temperature and time can have disrupted the cellular wall structure of the plant tissues, hence resulting in more antioxidant components such as free phenolics leached from the plant food (Shaharuddin et al., 2021; Wang et al., 2023). Moreover, the low oxygen percentage in the SHS oven/dryer and the inactivation of PPO due to the high temperatures might avoid the oxidation of phenolic compounds, resulting in an increase in them and in the antioxidant activity.

At the same time, contrasting results were reported in tomato fruit treated at 100°C for 4.5 min (Ceccanti et al., 2021). In this case, bound phenolic compounds, localized in the cell wall matrix of the plant cells and bound to structural proteins, cellulose, and pectin through covalent bonds via ether, ester, and carbon–carbon bonds, could be released and degraded through the disruption of the cell wall matrix, due to the high temperature and treatment duration (Shahidi & Yeo, 2016). Moreover, some phenolic acids and their conjugated forms can be converted from one type to another type during the processing (Wang et al., 2023), inducing a decrease in TPC.

Differently to boiling and conventional steaming, SHS treatment induced retention of ascorbic acid in tomato, spinach, artichoke, and mango (Ceccanti et al., 2021; Sehrawat et al., 2018) and of GLs and sulforaphane content (Chaisamlitpol et al., 2014; Lekcharoenkul et al., 2014), likely due to the almost absent oxygen in the SHS chamber.

Finally, although few studies reported the evaluation of carotenoids in plant food subjected to SHS thermal processing/drying, lycopene content was negatively affected by SHS (Ceccanti et al., 2021; Shaharuddin et al., 2021). The authors suggested that high temperatures can break the lycopene molecule into smaller fractions. Thus, the SHS temperature of 170°C used by Shaharuddin et al. (2021) might degrade the lycopene in the SHS‐treated Kejirak fruit extract. Nevertheless, despite the adverse outcomes observed in lycopene content due to the heating process, thermal treatment yielded processed tomatoes with enhanced stability and heightened lycopene bioavailability. In fact, the utilization of heat treatment facilitates the isomerization of lycopene, transitioning it from the all‐*trans*‐lycopene to *cis*‐lycopene. The consumption of *cis*‐lycopene may potentially improve its absorption into the lymphatic system when compared to its *trans* form Heredia et al. (2010).

### 2.3 Blanching

Blanching is a water‐based heat treatment technique widely used to enhance the shelf life of fruit and vegetables. This process involves immersing the food in hot water, typically within the temperature range of 70–100°C, for few seconds to several minutes (Chang et al., 2021; Huarte et al., 2021; Jiang et al., 2022; Luo et al., 2022; Managa et al., 2019; Mashitoa, Shoko, et al., 2021; Nambi et al., 2016; Ncube et al., 2022; Sicari et al., 2021). This thermal process constitutes an essential unit operation for maintaining product quality during long‐term storage periods, as it effectively deactivates enzymes such as PPO and peroxidases reducing the browning unpleased effects on polyphenol‐rich fruit and vegetables (Adetoro et al., 2021) and reduces the microbial load (Neves et al., 2019). Moreover, blanching is also commonly used for tomato peeling (Liu, Qu, et al., 2023; Rock et al., 2012). Table 4 reports a summary of works regarding blanching effects on bioactive compounds in plant foods.

**TABLE 4:** Effect of blanching on bioactive compounds in vegetables.

Plant species	Cooking conditions	Phytochemical changes	References
Beetroot (*Beta vulgaris*)	70, 75, 80, 85, and 90°C; 3, 6, 9, 12, and 15 min	↓TPC ↓Ascorbic acid ↑Antioxidant activity	Nambi et al. (2016)
Baby cabbage (*Brassica campestris* ssp*. chinensis*)	100°C; 30 s	= GLS ↓Myrosinase activity	Luo et al. (2022)
Chinese cabbage (*Brassica chinensis*)	95°C; 5 min	↑TPC ↑FRAP and TEAC ↑GLS (sinigrin)	Managa et al. (2019)
Cauliflower (*Brassica oleracea* var. *botrytis*)	100°C; 30 s	= GLS ↓Myrosinase activity	Luo et al. (2022)
White cabbage (*Brassica oleracea* var. *capitata*)	100°C; 30 s	= GLS ↓Myrosinase activity	Luo et al. (2022)
Red cabbage (*Brassica oleracea* var*. capitata* f. *rubra*)	100°C; 30 s	= GLS ↓Myrosinase activity	Luo et al. (2022)
Broccoli (*Brassica oleracea* var. *italica*)	100°C; 30 s	= GLS ↓Myrosinase activity	Luo et al. (2022)
Red radish root (*Raphanus sativus* var. *niger*)	100°C; 30 s	= GLS ↓Myrosinase activity	Luo et al. (2022)
Chinese cabbage (*Brassica rapa* ssp. *pekinensis*)	100°C; 30 s	= GLS ↓Myrosinase activity	Luo et al. (2022)
Green pepper (*Capsicum annuum*)	70, 75, 80, 85, and 90°C; 3, 6, 9, 12, and 15 min	↓TPC ↓Ascorbic acid ↓Antioxidant activity	Nambi et al. (2016)
Jute mallow (*Corchorus olitorius*)	82°C; 5 min	↑TPC ↑TFC ↑Antioxidant activity	Ncube et al. (2022)
Pumpkin (*Cucurbita moschata*)	95°C; 5 min (in plain water or in steam)	↓TPC in plain water blanching ↓TPC in steam blanching = ABTS in steam blanching ↓FRAP in steam blanching ↓FRAP and ABTS in plain water blanching	Mashitoa, Shoko et al. (2021)
Cardoon (*Cynara cardunculus* var. *altilis*)	98°C; 30 s	= TPC ↑TFC = DPPH ↓ABTS	Huarte et al. (2021)
Tiger nuts (*Cyperus esculentus*)	90°C; 5, 10, and 15 min	↑TPC ↓Antioxidant activity	Djikeng et al. (2022)
Cliff radicchio (*Hyoseris lucida* subsp. *taurina*)	90°C; 10 min	↓TCC ↓TPC ↓TFC ↓β‐Carotene ↓FRAP ↑DPPH and ABTS	Sicari et al. (2021)
*Hyoseris radiata*	90°C; 10 min	↓TCC ↓TPC ↓TFC ↓β‐Carotene ↓FRAP ↑DPPH and ABTS	Sicari et al. (2021)
Cat's ear (*Hypochaeris laevigata*)	90°C; 10 min	↓TCC ↓TPC ↓TFC ↓β‐Carotene ↓FRAP ↑DPPH and ABTS	Sicari et al. (2021)
Deep rooted catsear (*Hypochaeris radicata*)	90°C; 10 min	↓TCC ↓TPC ↓TFC ↓β‐Carotene ↑FRAP, DPPH, and ABTS	Sicari et al. (2021)
Green pea (*Lathyrus oleraceus*)	70, 75, 80, 85, and 90°C; 3, 6, 9, 12, and 15 min	↓TPC ↓Ascorbic acid ↓Antioxidant activity	Nambi et al. (2016)
Drumstick (*Moringa oleifera*)	100°C; 2 min	↓β‐Carotene ↓Ascorbic acid	Kachhawa and Chawla (2024)
White radish (*Raphanus sativus* var*. longipinnatus* Bailey)	100°C; 30 s	= GLS ↓Myrosinase activity	Luo et al. (2022)
Chayote (*Sechium edule*)	100°C; 2 min	↓TPC and TFC ↑DPPH ↑β‐Carotene	Chang et al. (2021)
Eggplant (*Solanum melongena*)	70, 75, 80, 85, and 90°C; 3, 6, 9, 12, and 15 min	↓TPC ↓Ascorbic acid ↓Antioxidant activity	Nambi et al. (2016)
*Undaria pinnatifida*	100°C; 3 min	↓TPC	Jiang et al. (2022)

*Note*: Plant food species are in an alphabetical order, considering the species Latin name.

Abbreviations: ABTS, 2,2′‐azino‐bis(3‐ethylbenzothiazoline‐6‐sulfonic acid) antioxidant activity assay; DPPH, 2,2‐diphenyl‐1‐picrylhydrazyl antioxidant activity assay; FRAP, ferric reducing‐antioxidant power assay; GLS, glucosinolates; TAC, total anthocyanin content; TCC, total carotenoid content; TFC, total flavonoid content; TPC, total phenolic content.

Various works reported a reduction in terms of TPC, TFC, and antioxidant activity in fruit and vegetables subjected to blanching treatment (Chang et al., 2021; Jiang et al., 2022; Nambi et al., 2016; Sicari et al., 2021), but results are conflicting (Djikeng et al., 2022; Huarte et al., 2021; Managa et al., 2019; Ncube et al., 2022). For example, in a quite recent study (Chang et al., 2021), blanching of *Sechium edule* shoots was performed by immersing them in boiling water at 100°C for 2 min. These authors observed a significant reduction in TPC (−32.1%) compared to the raw shoots, even though no significant differences were reported in terms of antioxidant activity. Moreover, the effect of the blanching method in *Beta vulgaris*, *Lathyrus oleraceus*, *Solanum melongena*, and *Capsicum annuum* was evaluated at different temperatures (70, 75, 80, 85, and 90°C) for 3, 6, 9, 12, and 15 min (Nambi et al., 2016). This study showed that TPC was subjected to a decrease that proceeded linearly with the increase of temperature and time (Nambi et al., 2016). As observed for the boiling process, these reductions indicate that blanching leads to the release of phenolic compounds stored in the cellulose network present in the cell wall in the water (Gunathilake et al., 2018). Notably, this treatment reported a lower percentage of TPC (67.9) retention in comparison to other thermal processes such as microwaving, stir‐frying, and steaming, which exhibited over 80% TPC retention (Chang et al., 2021). In this context, the use of blanching in plain water was compared with the use of water steam to blanch pumpkin leaves (Mashitoa, Shoko, et al., 2021). These authors found the highest retention of TPC using steam blanching, reducing the loss of rutin (−3.0%), kaempferol 7‐neohesperidoside (−7.4%), isoorientin 2″‐*O*‐rhamnoside (−10.6%), isorhamnetin‐3‐*O*‐rutinoside (−10.9%), quercetin 3‐galactoside (−14.8%), coumaroyl glucaric acid (−16.1%), isorhamnetin‐3‐galactoside‐6″‐rhamnoside (−16.7%), 2‐caffeoylisocitric acid (−18.8%), and quercetin 3‐galactoside 7‐rhamnoside (−23.1%), whereas coumaroyl isocitrate increased by 14.9%.

Conversely, a significant increase in terms of TFC (+73.2%) in blanched cardoon stalks was reported, even though nonsignificant differences were observed in terms of TPC and antioxidant activity evaluated using DPPH assay and an increase when evaluated using ABTS assay after a blanching process at 98°C for 30 s (Huarte et al., 2021). Another study reported an increase in TPC in Chinese cabbage leaves subjected to blanching at 95°C for 5 min (+26.0% compared to the raw samples), and, consequently, the antioxidant activity evaluated using both the FRAP and Trolox equivalent antioxidant assays reported higher values (+54.5% and +76.7%, respectively) than raw samples (Managa et al., 2019). According to the previous mentioned studies, a significant increase in TPC (+86.4%) and TFC (+43.7%), as well as in the antioxidant activity analyzed by DPPH (+23.8%), FRAP (+5.3%), and ABTS (+26.2%) assays, was observed in *Corchorus olitorius* leaves blanched at 82°C for 5 min compared to the raw leaves (Ncube et al., 2022). Different hypotheses have been formulated by some authors to explain these results (Juániz et al., 2016; Managa et al., 2019). The high temperatures used during the blanching process in a short time can induce a greater rupturing of cell structure, which would have led to better solvent access and phenolic compound extraction (Managa et al., 2019). Moreover, the thermostability of phenols and flavonoids has resulted in being dependent on their glycosylation and acylation *status*, and an increase in non‐acylated flavonoid compounds was already shown during thermal processing (Juániz et al., 2016; Managa et al., 2019). In addition, the disappearance of phenolic compounds such as protocatechuoyl‐hexose from Chinese cabbage subjected to blanching treatment as reported by Managa and coworkers could possibly have been due to the rupture of phenol–sugar bond, resulting in the formation of the simple phenolic structure of the aglycone and determining the reduction of TPC (Juániz et al., 2016; Managa et al., 2019).

The effect of blanching on the GLs content in Chinese cabbage leaves, especially the sinigrin content as the predominant GL present in *Brassica rapa* (Sun et al., 2019), was also analyzed (Managa et al., 2019). These authors observed an increase in sinigrin content (+19.0%) when compared to the raw leaves.

Moreover, high loss of ascorbic acid was observed in *L. oleraceus* and *S. melongena* (about 65%–70% in both species) and in *C. annuum* (about 50%) after a blanching treatment at 90°C for 15 min (Nambi et al., 2016). A similar result was reported for *Moringa oleifera* pods once blanched at 100°C for 2 min (Kachawa & Chawla, 2023). However, the high susceptibility of this molecule to high temperatures, independently to the time, is well known (Mieszczakowska‐Frac et al., 2021).

Concerning the effect of blanching on TCC, an increase in β‐carotene content was shown (Chang et al., 2021). Conversely, a significant decrease (−62.0%) in TCC was observed after blanching treatment at 90°C for 10 min on four leafy vegetables (*Hypochaeris laevigata*, *Hypochaeris radicata*, *Hyoseris radiata*, and *Hyoseris lucida* subsp. *taurine*) (Sicari et al., 2021). In support of these results, recently, it has been noted that blanching caused great loss in terms of β‐carotene (−49.2%) in *M. oleifera* pods (Kachawa & Chawla, 2023). β‐Carotene is a fat‐soluble vitamin; thus, its retention during thermal processes can be enhanced using hydrogenated fat (Kopec & Failla, 2018). Indeed, fat may solubilize the β‐carotene content of leafy vegetables, resulting in lower loss of TCC and β‐carotene than during blanching in hot water (Sicari et al., 2021).

### 2.4 Microwaving

Microwaving is a heat treatment commonly used domestically because of its ability to achieve high heating rates, reduction in time, safe handling, and ease of operation (Mehmood & Zeb, 2020). Several studies have investigated the microwaving effects on vegetable bioactive compounds, comparing this thermal process with boiling and steaming methods. Table 5 summarizes the recent scientific literature about the effect of microwaving on bioactive compounds in fruit and vegetables.

**TABLE 5:** Effect of microwaving on bioactive compounds in vegetables.

Plant species	Cooking conditions	Phytochemical changes	References
Peanut (*Arachis hypogaea*)	500 W; 3 min	↓TPC ↓TFC ↓DPPH, ABTS, and FRAP	Zhang et al. (2023)
Chinese kale (*Brassica alboglabra*)	900 W; 1, 2, and 3 min	↑TPC ↑DPPH and ABTS	Chin et al. (2022)
Mustard (*Brassica juncea*)	900 W; 1 min	↓Ascorbic acid ↑β‐Carotene = TFC = TPC = DPPH	Rana et al. (2021)
Broccoli (*Brassica oleracea* var. *italica*)	850 W; 5 min	↓TPC ↓DPPH	Czarnowska‐Kujawska et al. (2022)
Kale (*Brassica oleracea* var. *acephala*)	1000 W of power; 5 min	↓Soluble polyphenol content = Hydrolyzable polyphenol content = DPPH	Dolinsky et al. (2016)
Cabbage (*Brassica oleracea* var. *capitata*)	1000 W of power; 5 min	= Soluble polyphenol content ↑Hydrolyzable polyphenol content ↑DPPH	Dolinsky et al. (2016)
	900 W; 1 min	↓Ascorbic acid ↑β‐Carotene = TFC ↑TPC = DPPH	Rana et al. (2021)
Broccoli (*Brassica oleracea* var. *italica*)	1000 W of power; 5 min	= Soluble polyphenol content = Hydrolyzable polyphenol content = DPPH	Dolinsky et al. (2016)
	300 W; 8 min	↑TCC (in organic cultivation)	de Castro et al. (2021)
	700 W; 2 and 4 min	↑TPC = TFC ↑ABTS and DPPH ↓Sulforaphane	Kim et al. (2021)
	950 W; 19 min	↑DPPH = TPC ↑Ascorbic acid = GLS	Tabart et al. (2018)
Broccoli (*Brassica oleracea* var. *italica*)	800 W, 3 and 5 min	= GLS after 3 min and ↓GLS after 5 min = HCAs after 3 min and ↓HCAs after 5 min ↓DPPH	Paulsen et al. (2021)
Broccoli (*Brassica oleracea* var. *italica*)	600 W; 1, 3, and 5 min	↑TPC (after 5 min) = Ascorbic acid = ABTS and FRAP ↓DPPH (except after 3 min)	López‐Hernández et al. (2022)
Red cabbage (*Brassica oleracea* var. *capitata* f. *rubra*)	950 W; 19 min	= DPPH = TPC ↑Ascorbic acid ↓TAC = GLS	Tabart et al. (2018)
Quinoa (*Chenopodium quinoa*)	700 W; 2 min	↑TPC ↑TFC ↑DPPH and ABTS	Zhang et al. (2022)
	900 W; 2, 3.5, and 5 min	↑TPC ↑TFC (except at 5 min) ↑DPPH ↓ABTS (except at 3.5 min) = FRAP (except at 5 min)	Sharma et al. (2022)
Pumpkin (*Cucurbita moschata*)	900 W; 10 min	= TPC ↓Gallic acid, protocatechuic acid, vanillic acid, ellagic acid ↑Syringic acid, *p*‐coumaric acid, ferulic acid ↓ABTS ↑DPPH	Mashitoa, Manhivi et al. (2021)
	300 W; 12 min	↑TCC (in organic cultivation)	de Castro et al. (2021)
Squash (*Cucurbita* spp.)	1000 W of power; 5 min	= Soluble polyphenol content = Hydrolyzable polyphenol content ↑DPPH	Dolinsky et al. (2016)
Carrot (*Daucus carota*)	1000 W of power; 5 min	= Soluble polyphenol content = Hydrolyzable polyphenol content = DPPH	Dolinsky et al. (2016)
	300 W; 10 min	↓TCC	de Castro et al. (2021)
Green bean (*Lablab purpureus*)	900 W; 1 min	↓Ascorbic acid ↑β‐Carotene = TFC ↑TPC ↑DPPH	Rana et al. (2021)
African pumpkin (*Momordica balsamina*)	900 W; 15 min	↓Phenolic acids and flavonoids ↑Lutein ↓Zeaxanthin ↓β‐Carotene = TCC ↑DPPH and ABTS	Mashiane et al. (2022)
*Pereskia aculeata*	1.700 W; 5 min	↑TCC = TPC ↓Ascorbic acid ↑ORAC	Neves et al. (2021)
Green beans (*Phaseolus vulgaris*)	1000 W of power; 5 min	= Soluble polyphenol content ↓Hydrolyzable polyphenol content ↑DPPH	Dolinsky et al. (2016)
Pohpohan (*Pilea trinervia*)	450 W; 1 and 3 min	↓TPC ↑DPPH ↑Ferulic and caffeic acid	Ardiansyah et al. (2023)
Tomato (*Solanum lycopersicum*)	1000 W of power; 5 min	= Soluble polyphenol content ↓Hydrolyzable polyphenol content ↓DPPH	Dolinsky et al. (2016)
	1000 W, 30 and 300 s	↑TPC (after 300 s) ↑TFC (after 300 s) ↑Lycopene = DPPH and FRAP	Mahieddine et al. (2018)
Potato (*Solanum tuberosum*)	750 W; 7 min	↓Ascorbic acid ↑TPC ↑DPPH	Narwojsz et al. (2020)
Spinach (*Spinacea oleracea*)	850 W; 5 min	↓TPC ↓DPPH	Czarnowska‐Kujawska et al. (2022)
	850 W; 3, 6, 9, 12, and 15 min	↑β‐Carotene (after 9 and 15 min) ↑Violaxanthin (after 6, 9, and 15 min) ↑Lutein (after 9 and 15 min) ↑TPC ↑TAC ↑TFC (increasing with time) ↓DPPH (increasing with time, after 12 and 15 min)	Mehmood and Zeb (2023)

*Note*: Plant food species are in an alphabetical order, considering the species Latin name.

Abbreviations: ABTS, 2,2′‐azino‐bis(3‐ethylbenzothiazoline‐6‐sulfonic acid) antioxidant activity assay; DPPH, 2,2‐diphenyl‐1‐picrylhydrazyl antioxidant activity assay; FRAP, ferric reducing‐antioxidant power assay; GLS, glucosinolates; HCA, hydroxycinnamic acids; ITC, isothiocyanates; ORAC, oxygen radical absorbance capacity assay; TAC, total anthocyanin content; TCC, total carotenoid content; TFC, total flavonoid content; TPC, total phenolic content.

Most studies reported a retention of phenolic compounds and antioxidant activity in microwaved vegetables when compared with raw ones (Ardiansyah et al., 2023; Chin et al., 2022; Dolinsky et al., 2016; Kim et al., 2021; Mahieddine et al., 2018; Mashitoa, Manhivi, et al., 2021; Narwojsz et al., 2020; Neves et al., 2021; Rana et al., 2021; Sharma et al., 2022; Tabart et al., 2018; Zhang et al., 2022). For example, microwaving caused a maintenance of soluble polyphenol content, hydrolyzable polyphenol content, and antioxidant activity assayed by DPPH in several fruit and vegetables such as squash, broccoli, carrot, kale (except for soluble polyphenol content), cabbage, tomato (except for hydrolyzable polyphenol content and antioxidant assay), and green beans (except for hydrolyzable polyphenol content) treated for 5 min at a power of 1000 W (Dolinsky et al., 2016). Moreover, a significant increase in TPC and antioxidant activity was observed in microwaved broccoli for 2 and 4 min at 700 W (Kim et al., 2021). A study on the effect of microwave thermal process on tomatoes reported an increase of TPC and TFC after a treatment of 30 and 300 s (+3.62‐fold and +4.57‐fold, respectively) at 1000 W, highlighting that higher treatment duration led to an enhancement of phenolic compounds as the bioavailability of these bioactive compounds was positively correlated with the microwave treatment duration (Mahieddine et al., 2018).

Conversely, two recent studies reported opposite results, noting a decrease of TPC and antioxidant activity in peanut sprout and spinach leaves, respectively, microwaved at 850 W, a lower power level than that used by authors who observed an increase of TPC when they compared microwaved plant samples with raw ones (Czarnowska‐Kujawska et al., 2022; Zhang et al., 2023). The definition of temperature in microwaving is difficult to achieve, and it is strictly dependent on the water content of the edible plant portion (Datta & Rakesh, 2013). Indeed, most of the studies insert fruit and vegetables in water before allowing them to undergo microwaving (Chin et al., 2022; Czarnowska‐Kujawska et al., 2022; Dolinsky et al., 2016; Mahieddine et al., 2018; Mashiane et al., 2022; Mashitoa, Manhivi, et al., 2021; Neves et al., 2021). These results suggest the possible destruction of cell compartments and better extraction of polyphenols using 900 or 1000 W than 850 W. In addition, in the microwave treatment at 850 W, polyphenols could be leached in the water as already described in the boiling process. Another explanation of the increase in antioxidant compounds is the possible bond cleavage with other molecules such as proteins and sugars, thus increasing their availability during their extraction and quantification (López‐Hernández et al., 2022). However, the observed discrepancies could be also due to variations in extraction methods and the size of vegetables subjected to heat treatments.

Ascorbic acid degrades easily during thermal processing, and this is also confirmed in studies that evaluated the microwaving effect on the retention of bioactive compounds in vegetables (Narwojsz et al., 2020; Neves et al., 2021; Rana et al., 2021). However, contrasting results about the retention of ascorbic acid in vegetables subjected to microwaving are reported. For example, ascorbic acid increased significantly after treatment in both broccoli and red cabbage (+97% and +57%, respectively, compared to unheated samples) microwaved for 19 min at 950 W (Tabart et al., 2018). These authors hypothesized that, likely, during microwaving no cell destruction happens and that ascorbic acid diffusion out of cells could not be as rapid as other solutes (Howard et al., 1999; Tabart et al., 2018).

GLs content was retained in red cabbage and broccoli microwaved for 9 min at 950 W (Tabart et al., 2018) and for 3 min at 800 W (Paulsen et al., 2021). Moreover, the use of microwaving induced the breakage of GLs, determining an increase in ITCs such as sulforaphane, with an important antioxidant activity (Kim et al., 2021).

Interestingly, the microwaving induced, in most of cases, the increase/retention of TCC and some specific carotenoids such as lycopene, β‐carotene, lutein, and violaxanthin (De Castro et al., 2021; Mahieddine et al., 2018; Mashiane et al., 2022; Mehmood & Zeb, 2023; Neves et al., 2021; Rana et al., 2021). For instance, a significant 5.52‐ and 7.05‐fold increased lycopene content was shown in tomatoes microwaved at 1000 W for 30 or 300 s, respectively (Mahieddine et al., 2018). An increased β‐carotene content in cabbage, mustard, and green bean microwaved for 1 min at 900 W was also observed (Rana et al., 2021). Similarly, an increased β‐carotene, violaxanthin, and lutein content were noted in spinach leaves microwaved at 850 W for 9 and 15 min (Mehmood & Zeb, 2023). An increase in TCC by 12.1% was reported in microwaved *Pereskia aculeata* leaves (Neves et al., 2021). These authors also found increased *cis*‐isomer carotenoid content after microwaving (+19.0%) compared with raw samples. Moreover, they observed that TCC is proportional to temperature and duration, which corroborates their results as microwaving has a shorter time than the other thermal processes assessed in the study (boiling, steaming, and stir‐frying), leading to enhanced TCC not only when compared to raw samples but also when compared to samples heated with other methods (Neves et al., 2021).

## 3 BIOACTIVE COMPOUND BIOACCESSIBILITY AFTER THERMAL PROCESSING

From the nutritional standpoint, bioaccessibility is the fraction of an ingested compound that is available for absorption in the gut, and their consequent bioavailability is the fraction of an ingested compound that reaches the systemic circulation and tissues to exert its biological action for the organism (Polia et al., 2022), as it is discussed in the next section. Before becoming bioavailable, any bioactive compounds must be released from the food and eventually modified in the gastrointestinal tract. Bioaccessibility includes digestive transformations of food into material ready for absorption through gastric and intestinal epithelium cells, becoming then available for metabolism of the organism (Galanakis, 2021).

In most cases, the bioactive compounds extensively described in the previous section exert their antioxidant, anti‐inflammatory, vasoprotective, and cardioprotective properties, after their gastric and intestinal absorption. Therefore, understanding the mechanisms and efficiency of such absorption is of the utmost importance to depict more accurately the consequent effects on human health. For example, the absorption process of carotenoids, which are highly lipophilic, is very poorly understood, and we know that they are weakly absorbed. Thus, in this case, the use of adequate experimental models, mimicking the human intestinal digestion and absorption processes, would clarify the mechanism of modifications and absorption of such compounds (Reboul, 2019). Due to the highly invasive and economically and ethically expensive methods of analysis in in vivo settings, this approach represents a reliable and predictive system even for human studies.

Several in vitro digestion models have been developed to simulate the human gut and investigate the digestion of ingested foods (Sensoy, 2021), ranging from single to multiple‐compartment, static to semi‐dynamic, or fully dynamic systems to better simulate digestion in the oral cavity, stomach, and intestine (Wojtunik‐Kulesza et al., 2020).

In this context, several studies reported the use of in vitro digestion simulation to analyze the bioaccessibility of various compounds (Bohn et al., 2018).

In this section, we will discuss the bioavailability studies, in vitro and in vivo, of compounds such as carotenoids, polyphenols, and ascorbic acid, after thermal processing. The reported in vitro systems were chosen for their predictability and reliability in mimicking human digestion and absorption and for being a valid support and pillar for in vivo studies.

Concerning carotenoids, some authors demonstrated the increase in their bioaccessibility starting from carrots. These findings report that chopping and homogenizing the carrot significantly increased the release of carotenoids as well as thermal processing (Hedren et al., 2002; Tydeman et al., 2010). Ryan et al. (2008) analyzed the bioaccessibility of carotenoids through in vitro digestion and tested the intestinal absorption through an in vitro intestinal epithelium model. The authors focused on the micellarization of carotenoids from heated and raw plant samples, reporting that thermal processing did not have a significant effect, thus ensuring the absorption of lutein. In the same work, the authors also focused on the development of an in vitro digestion method to evaluate the bioavailability of carotenoids in meals. This study highlights the greater solubilization of lutein compared to other carotenoids during digestion, with bile and pancreatin playing crucial roles in the process. Liu et al. (2004) compared the absorption of carotenoids from pure compounds versus whole foods using a similar approach composed of in vitro digestion and intestinal absorption. The study examined the uptake of β‐carotene, zeaxanthin, and lutein. Results indicated that the cellular uptake of β‐carotene and zeaxanthin was higher than that of lutein. Importantly, dietary carotenoids from whole foods were absorbed similarly to pure compounds. The findings obtained by means of these in vitro intestinal models were further supported by observations in volunteering ileostomy patients (Livny et al., 2003). In this work, the results indicated that thermal processing leads to a significant increase in absorption of carotenoids as compared to the same raw food. On the other hand, with a similar approach, Tydeman et al. (2010), focusing on the ileal effluent samples and not on the global intestinal absorption, observed that the retention of carotene was mainly due to the resistance of the cell wall to be degraded by thermal processing or by upper gut digestion. In another study, Cardinault et al. (2003) recruited 16 volunteering healthy adults (8 young men and 8 old men) to whom vegetables rich in carotenoids, heated carrot puree (β‐carotene), heated tomato puree (lycopene), and heated chopped spinach (lutein) were given. The authors compared the postprandial chylomicron responses of β‐carotene, lycopene, and lutein between young and older subjects. They revealed that lycopene bioavailability was compromised in older individuals, whereas the bioavailability of the other carotenoids did not show significant age‐related differences. Although the results of this study referred to the carotenoids absorption, the thermal processing effects on bioavailability can be considered a secondary outcome among the results of the study, the first variable considered being the age‐related absorption.

Due to the mentioned low absorption rate of carotenoids, the combination of thermal processing with food additives can positively impact the bioaccessibility and bioavailability of carotenoids, and lipids are often tested as additives. Rosul et al. (2022) discussed the evaluation of carotenoids from different edible plants, comparing the bioaccessibility of carotenoids from formulated products containing different types of fat. They used vacuum‐dried pumpkin puree as a source of carotenoids and evaluated how different lipids (butter, sunflower oil, and coconut oil) impact the absorption of α‐carotene, β‐carotene, and lutein. In their approach, fats are added as ingredients in porridge, cookies, and sponge cake. The foods were in vitro digested, and the fats were separated in the form of micelles from the rest of the digestate. Lutein displayed the highest bioaccessibility as compared to α‐ and β‐carotene, and micelles from cookies (containing butter) led to the highest percentage of α‐carotene cell uptake as compared to other baked products. These findings were further supported by a quite recent study, focusing on the comparison of the effects of different lipases on carotenoids‐containing vegetables (tomato juice, spinach, and carrot juice) with the addition of peanut oil. Such addition significantly improved the bioaccessibility of total carotenoids in all tested samples (Iddir et al., 2021). Furthermore, Priyadarshani and Chandrika (2007) demonstrated the improved bioaccessibility of carotenoids in heated non‐leaf vegetables from Sri Lanka. Heat treatment increased the release of β‐carotene, further enhanced by the fats as additive to the food such as coconut milk. These kinds of evaluations and measurements have also been performed in humans, although with no information on the thermal processing of the food and vegetables, as reported in the study conducted by Tyssandier et al. (2003). The authors evaluated the bioavailability of the main dietary carotenoids—β‐carotene, lycopene, and lutein—directly in the human gastrointestinal tract, through nasogastric tubes delivering vegetables in the stomach and harvesting stomach and duodenal fluids. The 10 healthy male volunteers consumed tomato puree, carrot puree, and chopped spinach, with sunflower oil as a food additive. The authors reported, for the first time in vivo, the poor bioavailability of carotenoids. In particular, the limited transfer of the carotenoids from their vegetable to the micelles in the stomach is associated with reduced bioavailability. Although the study reports interesting and novel findings, the procedure to measure gastric and intestinal absorption appears to be highly invasive and confirmed already established in vitro data.

Concerning the polyphenols, in a very recent work, onion was used as a source of phenols subjected to different thermal processes (Cattivelli et al., 2023). The authors reported that heat treatment was associated to the highest amount of phenolics extractability during in vitro digestion. However, they also noticed that colonic fermentation of raw onions caused a significant increase in beneficial bacteria, particularly *Lactobacillales* and *Clostridia*, suggesting how the evaluation of effects due to thermal processing and/or food additives should be considered in a broader setting.

The influence of thermal processes on the in vitro bioaccessibility of phenols was also evaluated (Avila et al., 2023; Sans et al., 2019). The study by Sans and coworkers reported a significant increase in TPC after the gastric phase in different onion varieties as compared to their raw counterparts. On the other hand, the second report demonstrated how steaming, instead of other thermal processes, was able to facilitate the release of phenols from red cabbage, but all the thermal processes led to a reduction of phenols antioxidant activity as compared to corresponding raw foods. Some food additives were also evaluated for their ability to increase the bioavailability of polyphenols. For example, the modulation of spinach phytochemical recovery during digestion using lemon juice formulations was examined (Vujcic Bok et al., 2022). The results showed that adding lemon juice to spinach formulations increased the levels of various bioactive compounds, including polyphenols, flavonoids, and phenolic acids. High bioaccessibility of polyphenols and ascorbic acid during digestion was observed in almost all spinach and lemon juice formulations. Furthermore, spinach formulated with 20% lemon juice has shown potential as a source of dietary polyphenols with antioxidant and antidiabetic activities. The study provided important insights into the potential health benefits of spinach and lemon juice formulations, particularly regarding the availability and stability of the bioactive compounds during digestion.

Focusing on ascorbic acid, Mori et al. (2012) examined the impact of multilayered cookware on the absorption of nutrients from vegetables. The results showed that thermal processing with multilayer cookware led to an increase in the absorption of nutrients, especially potassium and vitamins. This improvement was associated with increased antioxidant potential, as indicated by the significant increase in blood levels of ascorbic acid and β‐carotene. A further study evaluated the bioavailability of ascorbic acid from different food formulations and supplements (Gregory, 1993). The results suggested similar bioavailability of ascorbic acid across different sources, including tablets, orange juice, orange segments, and heated broccoli, with no apparent impact from heat treatment. In fact, although raw broccoli showed a slight reduction in bioavailability, this difference was considered negligible in typical mixed diets.

## 4 CARDIOVASCULAR EFFECTS OF BIOACTIVE COMPOUNDS PRESENT IN FRUIT AND VEGETABLES

**FIGURE 3:**
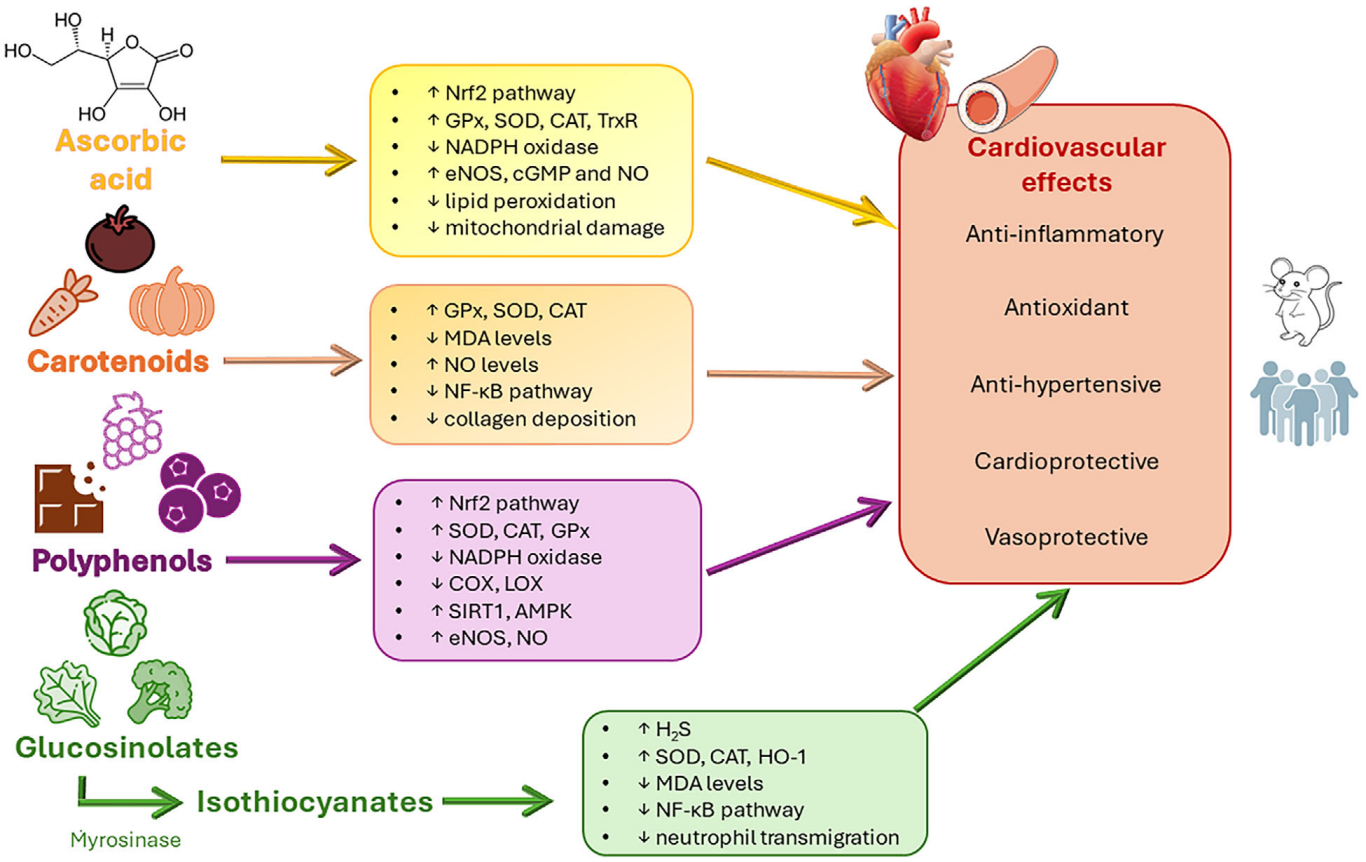
Cardiovascular effects of bioactive compounds present in fruits and vegetables. Ascorbic acid, carotenoids, flavonoids, and glucosinolates promote a variety of biological effects in the cardiovascular system, from anti‐inflammatory and antioxidant to antihypertensive and cardio/vasoprotective. This behavior results from the modulation of multiple signaling pathways. AMPK, adenosine monophosphate‐activated protein kinase; CAT, catalase; cGMP, cyclic guanosine monophosphate; COX, cyclooxygenase; GPx, glutathione peroxidase; H_2_S, hydrogen sulfide; HO‐1, heme oxygenase‐1; LOX, lipoxygenase; MDA, malondialdehyde; NADPH, nicotinamide adenine dinucleotide phosphate; NF‐κB, nuclear factor kappa‐light‐chain‐enhancer of activated B cells; NO, nitric oxide; Nrf2, nuclear factor erythroid 2‐related factor 2; SIRT1, sirtuin 1; SOD, superoxide dismutase; TrxR, thioredoxin reductase.

To date, very few studies focused on the impact of different thermal processes on the cardiovascular (CV) effects of fruit and vegetables. In a recent cohort study with a median follow‐up of 12 years, daily consumption of raw, but not heated, vegetables reduced the risk of CV disease and CVD mortality in nearly 400,000 adults (Feng et al., 2022). However, this work did not focus on specific vegetables but on “general” ones, including lettuce and tomato in sandwiches. As well known, the health benefits of fruit and vegetables depend heavily on those of nutraceuticals that, once absorbed, reach the bloodstream and interact directly with vascular cells, where they promote multiple biological properties. Therefore, the knowledge of the mechanisms and beneficial properties of isolated metabolites is crucial to fully understand the effects of fruit and vegetables on human health. In the next section, we will describe the antioxidant, anti‐inflammatory, antihypertensive, vasoprotective, and cardioprotective properties of specific bioactive compounds (ascorbic acid, carotenoids, polyphenols, GLs, and ITCs) (Figure 3), moving from preclinical to clinical studies, to provide a key to predicting the impact of thermal processes on the health effects of fruit and vegetables. This is a kind of “large” transitive property that could open new perspectives for future research: If thermal processing has an impact on the bioaccessibility and bioavailability of nutraceuticals, and if nutraceuticals have beneficial CV effects, then thermal processing could influence the CV effects of fruit and vegetables containing these nutraceuticals.

### 4.1 Ascorbic acid

#### 4.1.1 Radical scavenging activity and beyond

Ascorbic acid is the “king” of naturally occurring antioxidant agents. It is a powerful free radical scavenger that preferentially reacts with other radicals rather than non‐radical substances (Paciolla et al., 2019). Ascorbic acid is also a recognized activator of the antioxidant nuclear factor erythroid 2‐related factor 2 (Nrf2) and a stimulator of antioxidant enzymes, such as glutathione peroxidase (GPx), catalase (CAT), superoxide dismutase (SOD), and thioredoxin reductase (TrxR) (Gegotek & Skrzydlewska, 2023; Njus et al., 2020). Ascorbic acid exhibited antioxidant effects in the aorta of *ApoE*‐deficient mice (d'Uscio et al., 2003) and spontaneously hypertensive rats (SHRs), probably via activation of SOD and inhibition of the prooxidant enzyme nicotinamide adenine dinucleotide phosphate (NADPH) oxidase (Chen et al., 2001; Ulker et al., 2003). Ascorbic acid also mitigated the myocardial damage in a rat model of ventricular fibrillation by reducing oxidative stress, lipid peroxidation, and mitochondrial damage in the myocardium (Tsai et al., 2014, 2011) and alleviated oxidative stress in a rat model of cardiac toxicity (Hamza et al., 2022). In addition, ascorbic acid preserved the bioavailability of the vasorelaxant gasotransmitter nitric oxide (NO), which is synthesized by endothelial NO synthase (eNOS), in the vascular endothelium of *ApoE*‐deficient mice (d'Uscio et al., 2003). In this in vivo study, ascorbic acid also increased downstream levels of cyclic guanosine monophosphate (cGMP), further confirming the activation of the NO pathway (d'Uscio et al., 2003). A few years later, it has been shown that ascorbic acid significantly improved acetylcholine (Ach)‐induced relaxation of the aortic rings and attenuated the vasoconstrictor effect of phenylephrine (PE) in aorta of SHRs and diabetic rats. Worthy to note, the latter effect was significantly reduced in the presence of the eNOS inhibitor *N*ω‐nitro‐l‐argininemethyl ester (L‐NAME) (Ajay & Mustafa, 2006). Finally, a 6‐week treatment of SHRs with ascorbic acid prevented the hypertension progression, improved Ach‐induced vasorelaxation, and reduced aortic media‐to‐lumen ratio (Chen et al., 2001), whereas the incubation of ascorbic acid in the aorta isolated from SHRs restored eNOS activity, NO levels, and endothelial function (Ulker et al., 2003).

The potential anti‐inflammatory properties of ascorbic acid, which could further contribute to its biological effects in the CV system, are still unclear. Some studies have shown that consumption of ascorbic acid reduces plasma levels of C‐reactive protein (CRP) (Jialal & Singh, 2006), whereas others have shown that ascorbic acid has no effect on CRP (Colby et al., 2011). The lack of conclusive results is mainly due to the small number of studies evaluating the potential anti‐inflammatory effects of ascorbic acid alone, as it is generally in combination with other anti‐inflammatory compounds (e.g., carotenoids) (de Oliveira et al., 2012; Garcia‐Bailo et al., 2012).

#### 4.1.2 Ascorbic acid and CVDs: results from human studies

The possible association between dietary intake of ascorbic acid and reduced risk of CVDs, although widely investigated, is controversial. Several meta‐analyses have concluded that regular intake of ascorbic acid is associated with a lower risk of CVDs (Al‐Khudairy et al., 2017; Xu et al., 2022; Ye & Song, 2008) and CV mortality (Jayedi et al., 2019). Moreover, low serum concentrations of ascorbic acid are reported to be inversely associated with the subsequent incidence of stroke (Myint et al., 2008; Yokoyama et al., 2000). Many studies not included in the previous works, instead, showed no overall effect of ascorbic acid on major CV events, incidence of CVDs, and CV death in patients without a history of CVD (Cook et al., 2007; Forman et al., 2009; Mirmiran et al., 2022). Therefore, further studies are needed to clarify whether ascorbic acid intake could be effective in the prevention of CVDs. On the contrary, the possible role of ascorbic acid in patients with established CVD is much clearer. People with hypertension have low serum levels of ascorbic acid and, at the same time, ascorbic acid seems to be inversely associated with both systolic blood pressure (SBP) and diastolic blood pressure (DBP) levels (Ran et al., 2020). A meta‐analysis of randomized controlled trials (RCTs) showed that short‐term ascorbic acid supplementation (median dose: 500 mg/day, median intervention period: 2 months) significantly reduced SBP and DBP levels in patients with hypertension (change in SBP levels from the baseline: about −5 mmHg; change in DBP levels from the baseline: about −2 mmHg) (Juraschek et al., 2012). These findings were confirmed in a more recent meta‐analysis of clinical studies on the effects of ascorbic acid supplementation in patients with essential hypertension (Guan et al., 2020), where significative antihypertensive properties were found for intervention doses of ascorbic acid ≥500 mg and for a study duration ≥6 weeks. Besides direct BP‐lowering properties, ascorbic acid has also been demonstrated to promote beneficial effects in the human vascular endothelium. The results of a meta‐analysis of RCTs revealed a significant positive effect of ascorbic acid supplementation (>500 mg/day) on endothelial function measured by flow‐mediated dilation (FMD) (Ran et al., 2020).

These findings are consistent with those of other studies, where ascorbic acid improved endothelial function in patients with heart failure (Ashor et al., 2014), coronary artery disease (Gokce et al., 1999), and peripheral artery disease (Pleiner et al., 2008), indicating that regular intake of ascorbic acid could improve vascular function in patients with CVD.

### 4.2 Carotenoids

#### 4.2.1 Biological effects of carotenoids in the CV system: preclinical evidence

Carotenoids are hydrophobic compounds with recognized antioxidant effects, in part due to the ROS scavenging ability exhibited in lipophilic environments such as cell membranes (Rodriguez‐Concepcion et al., 2018). The different positions of polar carotenoids (e.g., zeaxanthin and lutein) and nonpolar carotenoids (e.g., carotenes and lycopene) within the bilayer have been shown to affect antioxidant potential of these molecules (Tapiero et al., 2004). For example, zeaxanthin effectively acts as antioxidant in the polar region of the membrane exposed to the aqueous environment, whereas carotenes and lycopene can quench radicals in the hydrophobic portion of the cell membrane (Tapiero et al., 2004). However, other mechanisms could contribute to the final effects of carotenoids in the CV system. Lycopene reversed the vascular aging process in the thoracic aorta of aged rats by reducing both aortic wall thickness and collagen deposition (Li et al., 2022). In a rat model of hyperhomocysteinemia (HHcy)‐induced atherosclerosis, daily lutein supplementation significantly prevented vascular oxidative stress by increasing the expression of SOD, GPx, and CAT in the aortic wall tissue (Wang et al., 2014). Furthermore, astaxanthin reduced ROS production in the aorta of SHRs (Chen et al., 2020; Monroy‐Ruiz et al., 2011), whereas lycopene prevented production of superoxide anion and increase of malondialdehyde (MDA) levels by enhancing the activity of SOD and GPx in the aorta of rats infused with the pro‐hypertensive agent angiotensin‐II (Ferreira‐Santos et al., 2018). In addition to vasoprotection, promising cardioprotective effects have also been reported for many carotenoids. Lycopene significantly reduced the left ventricular weight and the interstitial cardiac fibrosis in the heart of SHRs and angiotensin‐II‐infused rats (Ferreira‐Santos et al., 2018, 2021), whereas it improved the heart function in rats with isoproterenol‐induced myocardial infarction (Ojha et al., 2013). Moreover, astaxanthin delayed the incidence of stroke in stroke‐prone SHRs (Hussein et al., 2005), whereas β‐carotene increased the myocardial antioxidant capacity, limited the infarct size, and preserved the cardiac function in rat hearts subjected to I/R injury (Csepanyi et al., 2015). Furthermore, lycopene significantly reduced MDA levels, increased the activity of the antioxidant enzymes CAT, GPx, and SOD, and prevented morphological changes in the heart of rats with diabetes‐induced furan (Saracoglu et al., 2019). Of note, lutein suppressed pressure overload‐induced cardiac hypertrophy in rat hearts via activation of the regulator of ferroptosis interferon regulatory factor (Liu, Yang, et al., 2023). This finding, although preliminary, opens new perspectives in the pharmacology of carotenoids.

As previously described, CVDs are characterized not only by persistent oxidative stress but also by low‐grade inflammation. In the study by Wang et al. (2014), lutein significantly downregulated the expression of nuclear factor kappa‐light‐chain‐enhancer of activated B cells (NF‐κB) in the aorta of HHcy‐induced atherosclerotic rats.

Oxidative stress and inflammation are main features of atherosclerosis and hypertension. Worthy to note, lycopene prevented the development of atherosclerosis (Liu et al., 2021) and reduced the extent of atherosclerotic plaques and the intima‐media thickness in the aorta of *ApoE*‐deficient mice fed a high‐fat diet (Mannino et al., 2022). Furthermore, the same carotenoids but also astaxanthin exhibited promising antihypertensive properties in different rat models of hypertension (Abbasian et al., 2023). These effects could result from at least three different mechanisms: (i) vascular wall protection through antioxidant and anti‐inflammatory properties; (ii) arterial wall thickness/fibrosis reduction (Chen et al., 2020; Ferreira‐Santos et al., 2018; Hussein et al., 2006), and (iii) NO bioavailability increase. The NO‐mediated vasorelaxant effect of carotenoids, which have been previously described also for ascorbic acid, was demonstrated in many in vivo models of hypertension (Hussein et al., 2005; Monroy‐Ruiz et al., 2011; Sasaki et al., 2011), as well as in animal models of vascular dysfunction. In vivo, lutein increased NO bioavailability and reduced the levels of the vasocontracturant agent endothelin‐1 in the aorta of HHcy‐induced atherosclerotic rats, thus reversing endothelial dysfunction (Wang et al., 2014). Taken together, results of preclinical studies suggest that carotenoids could have a large impact on CV health.

#### 4.2.2 Potential benefits of carotenoids on CV health: results from human studies

Epidemiological studies have demonstrated that low dietary intakes of carotenoids have a role in the onset and progression of CVDs. A significant inverse association between lycopene intake and risk of coronary heart disease, stroke (Song et al., 2017), and hypertension (Li et al., 2019) has been demonstrated. More recently, high‐intake or high‐serum concentrations of lycopene were associated with reduced risk of stroke and mortality (Cheng et al., 2019). On the contrary, low plasma concentrations of lycopene and β‐carotene were significantly associated with carotid atherosclerosis in more than 200 asymptomatic patients, thus leading to hypothesize that regular intake of carotenoids might slow the onset and progression of atherosclerosis (Riccioni et al., 2008). The preventive effects exhibited by carotenoids against CVDs derive, in part, from their antioxidant properties. The results of a recent meta‐analysis showed that oral supplementation with carotenoids for more than 8 weeks stimulated the antioxidant defense system in healthy subjects, as demonstrated by a significant increase in both FRAP and oxygen radical absorbance capacity (Zhuang et al., 2022). Furthermore, lycopene supplementation (15 mg/day for 8 weeks) led to a significant increase in plasma SOD activity in healthy men (Kim et al., 2011). However, the beneficial properties promoted by carotenoids in the CV system are not limited to their antioxidant effects but, also, to the potential lipid‐lowering properties and the protective effects exhibited in the vascular wall. In this regard, lycopene restored vascular permeability and improved endothelial function in a subgroup of subjects with impaired endothelial function (lycopene dosage: 15 mg/day for 8 weeks) (Kim et al., 2011) or in patients with established CVD (lycopene dosage: 7 mg/day for 2 months) (Gajendragadkar et al., 2014). As known, progressive endothelial dysfunction and vascular damage can lead to the development of hypertension. Astaxanthin (8 mg/day for 8 weeks, orally) (Mashhadi et al., 2018) and crocin (15 mg/day for 12 weeks) (Behrouz et al., 2020) significantly reduced SBP levels in patients with type 2 diabetes. Similar results have been found for vegetables containing high levels of carotenoids, such as carrots and tomatoes. A recent meta‐analysis showed that consumption of carrots (100 g/day) could reduce the risk of hypertension (Madsen et al., 2023). However, this evidence derives from only three studies and must be confirmed.

The results of trials evaluating the CV benefits of tomato fruit, which is particularly rich in lycopene, are more consistent. Daily consumption of tomato fruit juice (200 g/day for 4 weeks; lycopene and β‐carotene contents after pasteurization at 80°C for 60 min: 16 ± 0.1 and 5.5 ± 0.0 ng/g, respectively) led to a marked decrease in SBP and to an increase of total antioxidant status in the serum of 13 patients with stage 1 hypertension (Michalickova et al., 2019). Regular intake of unsalted tomato juice (Nippon Del Monte; up to 700 mL/day for 12 months; lycopene content: 0.11 mg/mL) significantly reduced SBP and DBP levels in 260 Japanese subjects at risk of CVDs (Odai et al., 2019), whereas tomato paste puree (Berni; 80 g/day for 7 days; lycopene content unknown) significantly reduced DBP levels, increased brachial artery diameter, and decreased its stiffness after a fat meal in 19 healthy volunteers (Dalbeni et al., 2018).

Finally, high intake of raw tomatoes or tomato sauce (>100 g/day; lycopene dosage: 5.4 ± 3.4 mg/day) reduced hypertension risk by 36%, whereas moderate consumption of tomatoes (44–82 g/day; lycopene dosage: 2.1 ± 3.4 mg/day), both raw and processed, lowered blood pressure (BP) in patients with grade 1 hypertension (Murcia‐Lesmes et al., 2024). However, the data from patients who consumed raw tomatoes or tomato sauce were pooled and, therefore, it was not possible to distinguish the biological effects of the two products.

### 4.3 Polyphenols

#### 4.3.1 Effects of polyphenols in the CV system: results of preclinical studies

**FIGURE 4:**
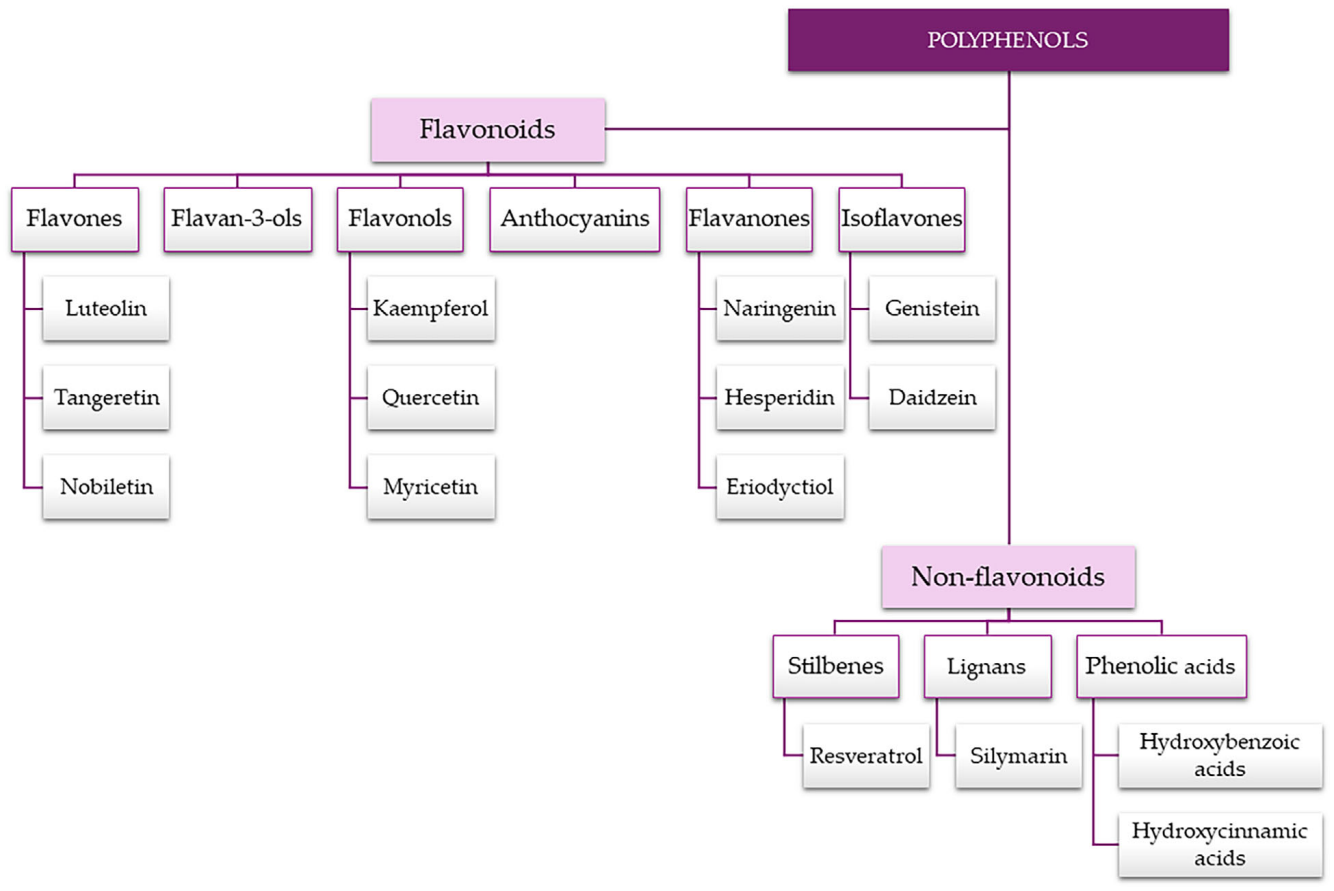
Classification of the main bioactive polyphenols.

Polyphenols are considered anti‐nutritional substances but exhibit health benefits in preventing CVDs (Behl et al., 2020). Polyphenols have a variety of structural forms, from simple molecules to polymerized ones, but their main structure is based on aromatic rings. Depending on their structure, this class could be divided into two major groups: flavonoids and non‐flavonoids and further subgroups (Figure 4).

Preclinical evidence demonstrated that polyphenols could prevent CVDs, including platelet aggregation, hypertension, endothelial dysfunction, and hyperlipidemia (Di Pietro et al., 2020; Yamagata, 2019). Polyphenols exert direct antioxidant effects due to the presence of hydroxyl groups, leading to the reduction of oxidative stress (Anwar et al., 2014; Fraga et al., 2010; Ziolkiewicz et al., 2023). These compounds show both a direct effect on free radicals (Pollard et al., 2006) and an indirect effect through activation of detoxifying enzymes such as CAT, SOD, and GPx. Polyphenols even reduce ROS production by inhibiting enzymes that generate free radicals such as NADPH oxidase or xanthine oxidase (Iqbal et al., 2023; Nijveldt et al., 2001). Finally, polyphenolic compounds activate Nrf2 (Hussain et al., 2022; Iranshahy et al., 2018) and sirtuin 1 enzyme (SIRT1) (Ciccone, Piragine, Brogi, et al., 2022; Testai et al., 2020; Xia et al., 2014), which plays a central role in the regulation of antioxidant, anti‐inflammatory, and antiaging signaling pathways (Ungurianu et al., 2023). A further effect triggered by SIRT1 is also the co‐activation of adenosine monophosphate‐activated protein kinase which, in turn, leads to activation of eNOS via phosphorylation and increases NO biosynthesis at endothelial level (Ungurianu et al., 2023; Xia et al., 2014). Xia et al. (2014) focused on resveratrol, which activates SIRT1 both through a direct mechanism by binding its regulatory domain and through an indirect mechanism by inhibiting phosphodiesterase enzymes. Another important property of polyphenols is the anti‐inflammatory activity. It is mainly mediated by the inhibition of production of pro‐inflammatory prostaglandins, leukotrienes, and thromboxane through blockade of cyclooxygenase (COX) and lipoxygenase (LOX) enzymes (Al‐Khayri et al., 2022; Garcia‐Lafuente et al., 2009; Iqbal et al., 2023; Serafini et al., 2010). This effect is mediated by the inhibition of NF‐κB which provokes the reduction of pro‐inflammatory mediators (Joo et al., 2018; Vitale et al., 2017; Ziolkiewicz et al., 2023).

Polyphenols show positive effects on endothelium integrity, improving FMD and reducing CV risk. They increase NO generation by enhancing Ca^2+^ intracellular concentration (Duarte et al., 2004; Fisher et al., 2003; Leikert et al., 2002; Martin et al., 2002; Ndiaye et al., 2005), and some of them also inhibit intercellular adhesion molecule‐1 (ICAM‐1)‐dependent monocyte adhesion and vascular adhesion molecule‐1 (VCAM‐1) (Dell'Agli et al., 2006; Di Pietro et al., 2020; Jiang et al., 2015; Lin, Ou, et al., 2015). In addition, polyphenols have anti‐atherosclerosis properties, reducing lipid buildup, neointimal growth, low‐density lipoprotein (LDL) oxidation, and increasing high‐density lipoprotein (HDL) content (Lin, Liu, et al., 2015; Loke et al., 2010; Xu et al., 2015). Preclinical studies on C57BL/6J mice, *ApoE*‐deficient mice, or Wistar rats fed with HF diet and daily treated with quercetin showed a reduction of total cholesterol (TC), triglycerides (TGs), and LDL together with an increment of HDL levels (Espirito‐Santo et al., 2023). The anti‐atherosclerotic effect was also observed by Kurosawa et al. (2005) in hypercholesterolemic rabbits receiving cacao flavonols for 6 months. Indeed, they observed a reduction of ox‐LDL and of atherosclerotic lesions area in aorta (Kurosawa et al., 2005). Furthermore, an antiplatelet action was observed in in vivo experiments, due to inhibition of COX and LOX enzymes, which reduced thromboxane A (TXA) synthesis, and a direct antagonism of the TXA2 receptor (Hamid et al., 2020; Iqbal et al., 2023; Loke et al., 2010; Olas et al., 2008).

Few studies have demonstrated that polyphenols or plant extracts rich in polyphenols treatment reduce BP and determine an endothelium‐dependent vasorelaxant effect in normotensive or hypertensive rats (Duarte et al., 2004; Espirito‐Santo et al., 2023). Martelli, Flori et al. (2021) demonstrated that the polyphenol‐rich extract named Taurisolo induced direct vasorelaxant effect on rat aortic rings, prevented the increase of BP, endothelial dysfunction, and cardiac hypertrophy in SHRs when administered chronically and, at clinical level, improved the endothelial function (measured as % FMD) in healthy volunteers.

#### 4.3.2 Protective role of polyphenols against CVDs: clinical evidence

Several clinical studies have explored the preventive role of polyphenols against CVDs. A diet rich in polyphenols exerts beneficial properties in the CV system, showing a reduction of BP, inflammatory markers, atherosclerosis process, and endothelial dysfunction (Karam et al., 2018; Ziolkiewicz et al., 2023). Growing evidence indicates that the Mediterranean diet promotes protective effects in the CV system, mainly due to the high content in polyphenols in plant foods (Guo et al., 2016; Tresserra‐Rimbau et al., 2014). Prevenciòn con Dieta Mediterranea, one of the largest observational studies exploring Mediterranean diet effects on CV events, involved 7169 Spanish individuals who have adhered to the Mediterranean diet for almost 5 years (Tresserra‐Rimbau et al., 2014). A long‐term polyphenol intake was reported to be associated with a reduction of risk of major CV events and mortality (Tresserra‐Rimbau et al., 2014). Many clinical trials also investigated the use of polyphenols or polyphenols‐rich products supplementation as anti‐CVD strategies. Citrus fruit and juices are a source of flavonoids among which flavanones, including hesperetin, hesperidin, naringenin, tangerine, and eriodictyol, account almost 95% of citrus fruit flavonoids (Chanet et al., 2012; Peterson et al., 2006). Experimental studies have demonstrated an inverse correlation between citrus flavanones consumption and the incidence of CVDs (Chanet et al., 2012; Mink et al., 2007).

Rizza and coworkers have conducted a placebo‐controlled double‐blind study on 28 individuals with metabolic syndrome who were supplemented with 500 mg hesperidin for 3 weeks. At the end of treatment, an improvement of FMD and a reduction of circulating inflammatory markers and plasma LDL and TC were reported (Rizza et al., 2011). The preventing role played by hesperidin was investigated also by Salden and coworkers who enrolled overweight and obese subjects treated with hesperidin 2S (500 mg/day) for 8 weeks. In this study, hesperidin determined a reduction of both DBP and SBP levels, leading to an important reduction of risk of mortality from coronary heart disease and stroke (Cook et al., 1995; Salden et al., 2016; Whelton et al., 2002). Furthermore, a slight reduction of VCAM‐1, ICAM‐1, and soluble P‐selectin was observed after the 6‐week treatment (Salden et al., 2016). In the study by Morand and coworkers, hesperidin, which is the predominant (90%) flavanone in the orange fruit, has been considered the responsible for the beneficial effect of orange juice intake. The reduction of BP provoked by daily ingestion of 500 mL of orange juice was comparable to the one induced by capsules containing the same dosage of hesperidin (292 mg) (Morand et al., 2011).

Daily supplementation with 400 mg naringenin, another citrus flavanone, for 8 weeks lowered plasma level of TC and increased the activity of antioxidant enzymes such as CAT or SOD in hypercholesterolemic patients (Jung et al., 2003). More recently, a clinical trial evaluated the effect of different citrus beverages (Rendeiro et al., 2016). In this study, middle‐aged healthy men randomly consumed an orange juice as control, a flavanone‐rich orange juice, and the whole orange in homogenized form after high‐fat meal ingestion. The reduction of FMD was counteracted by all the three flavanone interventions, and this effect is supposed to be correlated with a modulation of NO bioavailability (Rendeiro et al., 2016). Along with citrus fruit, bergamot was highly studied due to the high content of flavanols. Indeed, clinical trials reported that bergamot, consumed in different forms as tablets containing Bergamot Polyphenols Fraction, can reduce plasma LDL‐C and TC levels and increase HDL levels (Nauman & Johnson, 2019).

Moreover, berries such as blueberry, cranberry, strawberry, and blackberry are sources of anthocyanins which exert antioxidant properties. These fruits, such as blackcurrant or raspberry, contain phenolic acids as ferulic acid which is known for its antioxidant property (Sevgi et al., 2015). Daily intake of these fruits improved FMD in randomized clinical trials by Rodriguez‐Mateos et al. (2014, 2016, 2013). In 2019, a double‐blind RCT investigated the cardioprotective effect of a blueberry cup (150 g) daily intake in male subjects for 6 months (Curtis et al., 2019). The results revealed an increment of endothelial function, measured as FMD, and an improvement of systemic arterial stiffness and lipid profile. Therefore, a cup of blueberry was identified as an effective dietary supplementation to reduce CV risk (Curtis et al., 2019). In the study by Basu and coworkers, 48 subjects with metabolic syndrome consumed freeze‐dried blueberries for 8 weeks. Reduction of both SBP and DBP was observed along with an improvement of ox‐LDL levels (Basu et al., 2010). In a recent trial, 61 healthy volunteers received 26 g of freeze‐dried wild blueberry powder containing 302 mg of anthocyanins. This daily supplementation determined an increment of FMD by 0.85% and a reduction of SBP by −3.59 mmHg compared to placebo group (Wood et al., 2023). These results reflected the ones observed in a previous study in which an increment of 1.5% FMD and a reduction of SBP by 5.6 mmHg were reported (Rodriguez‐Mateos et al., 2019). Many other clinical studies demonstrated the preventive effect of this class of fruit and its polyphenolic constituents (Del Bo et al., 2014; Martini et al., 2023; Rousseau et al., 2021; Stote et al., 2020). Clinical trials also focused on the preventive effects of strawberries that are rich in anthocyanins. One month of strawberry supplementation (500 g/day) (Alvarez‐Suarez et al., 2014) or 6‐week consumption of strawberry freeze‐dried fruit (10 g/day) (Burton‐Freeman et al., 2010) determined a significant reduction of plasma LDL‐C, TC, and TG levels. Moreover, a protection against high carbohydrate/fat meal was observed in subjects receiving 10 g/day freeze‐dried fruit for 6 weeks (Ellis et al., 2011). Consumption of raspberries and ingestion of blackcurrant juice (250 mL/day) determined an increment of FMD level and an improvement of oxidative stress, endothelial dysfunction, and inflammation markers (Burton‐Freeman et al., 2010; Khan et al., 2014). The improvement of lipidic parameters was observed also in clinical trials based on anthocyanin supplementation (Broncel et al., 2007).

Among flavonols, quercetin is found in apples, tea, broccoli, onion, olives, blueberries, and red grapes, and it is used as supplementation due to its anti‐inflammatory, antioxidant, antitumoral, antiviral, and CV protective role (Popiolek‐Kalisz & Fornal, 2022; Shabir et al., 2022). Quercetin exerts beneficial properties in hypertensive conditions reducing BP and improving endothelial function. Several studies have demonstrated that treatment with quercetin reduced BP levels in hypertensive subjects and indicated a recommended dose of ≥500 mg/day in treatment period lasting ≥8 weeks (Dagher et al., 2021; Serban et al., 2016; Shabir et al., 2022). Of note, acute administration of 1095 mg quercetin in 12 hypertensive men (Larson et al., 2012) and chronic treatment with 730 mg quercetin for 28 days in prehypertensive and hypertensive men determined a reduction of BP levels (Edwards et al., 2007).

Isoflavones are another class of polyphenols widely present in leguminous plants. Soy and its derivatives are the major sources of isoflavones. Isoflavones are structurally like estrogens and are capable of playing pseudo‐hormonal activities interacting with estrogenic receptors (Behl et al., 2020). Daidzein, glycitein, and genistein are the main compounds (Krizova et al., 2019). Several epidemiological studies have demonstrated that soy derivatives or soy isoflavones can reduce CV risk. A 6‐month supplementation with 40 g soy flour or 63 g daidzein determined a reduction of LDL‐C (Liu et al., 2014). Of interest, 350 postmenopausal women without CVD and diabetes received 25 g of soy protein (containing 91 mg of isoflavones, divided into 52 mg of genistein, 36 mg daidzein, and 3 mg glycitein) or placebo for 2.7 years, and a nonsignificant reduction by 16% of carotid artery intima‐media thickness was observed (Hodis et al., 2011). The protection against CVDs was observed in several clinical studies. A total of 200 middle‐aged women daily ingested 15 g of soy protein with 66 mg isoflavones or soy protein alone as snack bars for 6 months (Sathyapalan et al., 2018). This supplementation reduced the 10‐year risk of coronary heart disease by 27%, myocardial infarction by 37%, and CVDs by 24% (Sathyapalan et al., 2018). Of interest, soy isoflavones exert beneficial effects in systemic and vascular inflammation and oxidative stress. Yari and coworkers enrolled 42 peritoneal dialysis patients who were supplemented with 100 mg isoflavone/day for 8 weeks, and, at the end of the treatment, a significant reduction of VCAM‐1 and ICAM‐1 was reported. No alterations in E‐selectin, MDA, and high‐sensitivity CRP levels were observed (Yari et al., 2020). In another study, soy isoflavones ameliorated BP homeostasis in women (Lu et al., 2020).

### 4.4 Glucosinolates and isothiocyanates

#### 4.4.1 CV benefits of natural sulfur compounds: the “hidden” role of hydrogen sulfide

**FIGURE 5:**
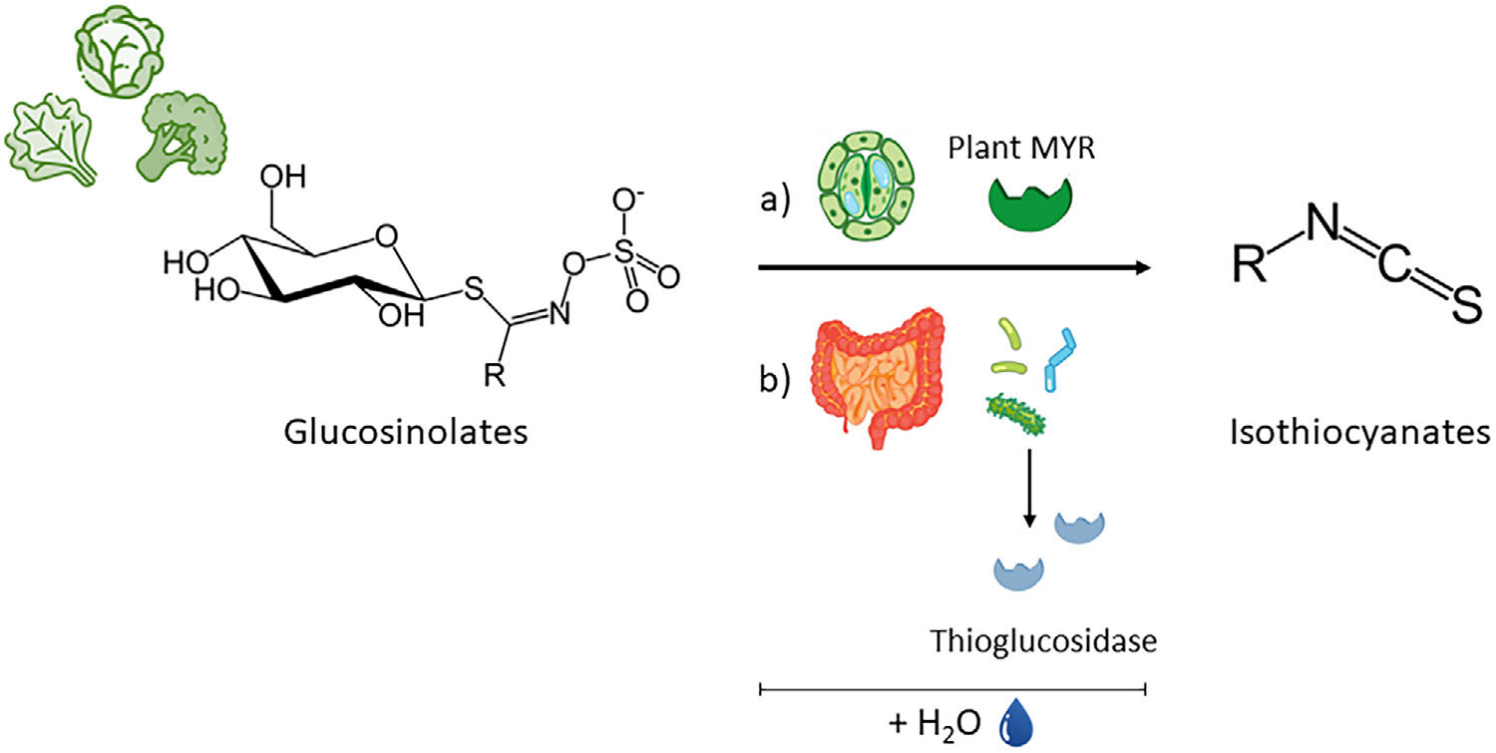
Reaction scheme of the hydrolysis of glucosinolates to isothiocyanates by (a) plant myrosinase (MYR) and (b) bacterial thioglucosidase in the human gut after chewing, processing or digestion of vegetables.

GLs are sulfur compounds present in plants of the *Brassicaceae* (or *Cruciferae*) family that, following chewing or food processing, are converted into the corresponding ITCs by the plant enzyme myrosinase. However, to a lesser extent, bacterial thioglucosidase can also hydrolyze GLs to ITCs in the human gut (Angelino et al., 2015) (Figure 5).

The pharmacology of GLs and ITCs has gained increasing interest, particularly after the discovery of the hydrogen sulfide (H_2_S)‐releasing properties of ITCs (Martelli et al., 2023). Although known for years exclusively as a toxic agent, H_2_S is now recognized as the third endogenous gasotransmitter, together with carbon monoxide (CO) and NO (Papapetropoulos et al., 2015). Under physiological conditions, H_2_S biosynthesis is tightly regulated and involves at least three enzymes, namely, cystathionine γ‐lyase, cystathionine‐β‐synthase, and 3‐mercaptopyruvate sulfurtransferase (Rose et al., 2017). Once produced, H_2_S rapidly crosses cell membranes, diffuses into many organs and tissues, and produces posttranslational modification (i.e., persulfidation) of several proteins, thus leading to changes in their structure. Mainly through persulfidation reactions, H_2_S: (i) opens sarcolemmal potassium channels in vascular smooth muscle cells (VSMCs) and directly promotes vasorelaxation, (ii) induces the nuclear translocation of Nrf2, thus activating the endogenous antioxidant machinery through the Nrf2/antioxidant response element pathway, (iii) exhibits anti‐inflammatory properties via inhibition of the nuclear factor NF‐κB, and (iv) participates in the modulation of the NO pathway (Szabo, 2018). However, current evidence indicates that the endogenous “H_2_S system” is compromised in patients suffering from the most common age‐related diseases, including CVDs (Piragine et al., 2023). Therefore, restoring the physiological concentration of the gasotransmitter with exogenous sources of H_2_S could represent an effective strategy to slow down the onset and progression of CVDs. In 2014, Citi and colleagues demonstrated that many natural ITCs, regardless of their chemical structure and origin, can slowly generate the gaseous molecule H_2_S in aqueous solution (Citi et al., 2014). A few years later, the same research group described the H_2_S‐releasing behavior of the dietary ITC erucin into biological substrates. Erucin, which derives from both the hydrolysis of glucoerucin (mainly found in *Eruca sativa* Mill., rocket salad or arugula) and the conversion of the ITC sulforaphane (from glucoraphanin, mainly present in *Brassica oleracea* L., broccoli) in vivo, slowly released H_2_S in rat cardiomyoblasts (i.e., H9c2) (Testai et al., 2023) and human vascular cells (Martelli, Piragine, et al., 2020; Martelli, Piragine, et al., 2021) in a concentration‐dependent manner. This interesting property could, in part, explain the protective effects exhibited by ITCs in the CV system. Indeed, there is an impressive overlap between the biological properties of H_2_S and ITCs, ranging from antioxidant and anti‐inflammatory to cardioprotective and vasoprotective roles (Martelli, Citi, et al., 2020). Sulforaphane reduced circulating adhesion molecules and monocyte‐derived macrophages, preserved elastin fiber organization, and maintained endothelial barrier function in the aorta of tumor necrosis factor‐α–treated mice (Nallasamy et al., 2014). In an in vivo mouse model of lipopolysaccharide‐induced peritonitis, intraperitoneal administration of the H_2_S‐donor erucin reduced neutrophil transmigration into the peritoneal cavity, which is a crucial step in the inflammatory cascade (Ciccone, Piragine, Gorica, et al., 2022). The antioxidant and anti‐inflammatory properties of natural ITCs could be also responsible for the promising cardioprotective effects demonstrated in preclinical studies. In fact, short‐term treatment with sulforaphane attenuated cardiac I/R injury by increasing SOD, CAT, and heme oxygenase‐1 levels in isolated rat heart (Piao et al., 2010), whereas chronic administration of sulforaphane prevented the increase in right ventricular systolic pressure, mitigated right ventricular hypertrophy and vascular remodeling, reduced the amount of the oxidative marker MDA, and increased the expression/activity of antioxidant enzymes (i.e., SOD) in the serum of mice exposed to chronic hypoxia (Pan et al., 2023).

As described above, the main risk factors for CVDs are atherosclerosis and hypertension. Benzyl ITC (from *Lepidium sativum* L., garden cress) showed vasorelaxant effects on PE‐contracted tissue strips (Wilson et al., 2002), whereas oral treatment with sulforaphane significantly reduced BP levels, arterial wall/lumen ratio, and number of VSMCs in the aorta of stroke‐prone SHRs (Senanayake et al., 2012). In another study, daily treatment with sulforaphane attenuated the development of atherosclerosis, improved vascular reactivity, and reduced the intima/media ratio in the aorta of hypercholesterolemic rabbits (Shehatou & Suddek, 2016). Also, the ITC erucin showed vasorelaxant properties in isolated rat aortic rings. In the absence of the endothelium or in the presence of the eNOS inhibitor L‐NAME, the vasorelaxant effects of erucin were markedly lower than those observed in intact aortic rings, leading to the hypothesis of a possible modulation of the endogenous “NO system” by the natural H_2_S‐donor. The potential antihypertensive effects of erucin were also demonstrated in in vivo models, where intraperitoneal administration significantly reduced SBP in SHRs but not in normotensive rats (Martelli, Piragine, et al., 2020). Similarly, *E. sativa* Mill. extracts standardized in GLs and ITCs promoted vasorelaxant properties and BP‐lowering effects in SHRs (Salma et al., 2018; Testai et al., 2022) and PE‐hypertensive rats, probably via H_2_S‐dependent effects (Testai et al., 2022).

#### 4.4.2 Cruciferous vegetables intake and CV outcomes: clinical evidence

About 10 years ago, Zhang and coworkers demonstrated an inverse association between consumption of cruciferous vegetables and reduced risk of CV mortality in more than 130,000 Chinese adults during a mean follow‐up period of 4.6 years for men and 10.2 years for women. Of note, the risk of mortality for CVDs decreased in parallel with the increase in vegetables intake, further confirming the proposed relationship (Zhang et al., 2011). More recently, a possible inverse association between regular intake of cruciferous vegetables (i.e., cabbage, brussels sprouts, cauliflower, and broccoli) and 15‐year atherosclerotic vascular disease (ASVD) mortality (e.g., ischemic heart disease mortality) has been observed in a cohort of 1226 older women. In the daily dose‐adjusted analysis, it was shown that per 10 g/day intake of cruciferous vegetables, there was a 12% lower risk of death due to ASVD (Blekkenhorst et al., 2017). Finally, results from three recent prospective cohort studies showed that regular intake (i.e., 1/2 cup for more than four times per week) of broccoli, but not cauliflower or Brussel sprouts, was associated with a reduced risk of incident hypertension in 190,000 subjects with more than 20 years of follow‐up (Borgi et al., 2016).

The main findings of epidemiological studies have been partially confirmed in clinical trials, but results are rather conflicting. A Phase 1 study investigated the effects of fresh broccoli sprouts (100 g/day for 1 week) on several biomarkers of lipid metabolism and oxidative stress in 12 healthy subjects (6 males and 6 females). A significant reduction of TC and LDL levels, as well as a marked increase of HDL levels, was observed at the end of the treatment only in the female group. Furthermore, a reduction in the levels of 8‐hydroxydeoxyguanosine, a marker of DNA damage, and an increase in the CoQ10H2/CoQ10 ratio (where CoQ10H2 is the first antioxidant against oxidized lipoproteins) was also reported (Murashima et al., 2004). In another study, 48 volunteers were asked to consume 2 different diets for 12 weeks and were randomly assigned to two groups: (i) broccoli‐rich diet (400 g/week of standard broccoli); (ii) broccoli rich in glucoraphanin (HG broccoli) (Armah et al., 2013). Dietary interventions did not lead to a reduction in either TC concentrations or BP levels in both groups. However, two phenotypes with different responses were identified in the HG broccoli group. These phenotypes were differentiated by single nucleotide polymorphisms associated with the poly(A) polymerase gamma gene (Armah et al., 2013), a poorly studied gene encoding for hsPAPγ protein that may regulate several hepatic genes (Thony et al., 1995). Within the HG broccoli group, the two genotypes had different levels of flavin adenine dinucleotide, a redox cofactor involved in fatty acid oxidation, which was higher in one genotype after treatment. This genotype had both downregulation of the expression of genes involved in mitochondrial respiration and increased citrate export from the tricarboxylic acid cycle, which results in high concentrations of fatty acids, lysolipids, and steroids (Armah et al., 2013). Two years later, the same authors published the results of two RCTs that enrolled 130 volunteers randomized to receive two dietary interventions (i.e., 400 g/week of standard broccoli or 400 g/week of HG broccoli for 12 weeks) to evaluate whether a diet rich in high levels of glucoraphanin can reduce plasma LDL levels. Although both diets were effective in reducing LDL levels, HG broccoli showed higher lipid‐lowering effects compared to standard broccoli, suggesting the key role of the GL glucoraphanin in the beneficial properties of broccoli sprouts (Armah et al., 2015). In contrast, Christiansen and coworkers did not observe beneficial effects of cruciferous vegetables in the CV system. They divided 40 hypertensive patients into two groups: One was the control group (normal diet) and the other received 10 g/day of dried broccoli sprouts for 4 weeks. At the end of the treatment period, no significant changes in BP levels, endothelial function, or blood lipid levels were reported. However, the drying process of broccoli sprouts may have reduced the concentration of intact GLs in the plant material (Christiansen et al., 2010). Other studies have explored the potential effects of broccoli in patients with CV risk factors, such as obesity and Type 2 diabetes. On the one hand, consumption of broccoli sprouts significantly decreased serum insulin levels and inflammatory markers (e.g., interleukin‐6 and CRP) but failed to reduce BMI in patients at high CV risk (Bahadoran et al., 2012; Lopez‐Chillon et al., 2019). In conclusion, current clinical evidence on the biological effects of cruciferous vegetables in the CV system is inconclusive, likely due to the heterogeneity of patient populations, experimental conditions, selection of plant material, and thermal processes. Further studies are needed to clarify the role of these plants in the prevention and management of CVDs. In this regard, an ongoing randomized controlled crossover trial enrolled 25 subjects with mildly elevated BP. The study, which confirms the growing interest in GLs‐rich plant foods, aims to compare the CV effects of a cruciferous plant soup (300 g/day) with a standard soup with the same energy, protein, fat, and carbohydrate content but without cruciferous vegetables (Connolly et al., 2020).

## 5 CONCLUSIONS

Most bioactive compounds found in fruit and vegetables have a positive impact on human health by reducing oxidative stress, inflammation, and the risk of chronic diseases such as cancer, CVDs, and metabolic disorders. Their consumption as part of a balanced diet is essential for the maintenance of good health and well‐being, especially in the weaker segments of the population and in developing countries, where food insecurity is a prevalent social determinant of health (Righettini & Bordin, 2023). The dietary intake of fruit and vegetables, however, is not the only aspect to be considered for optimal nutrition. In fact, many fruit and vegetables (e.g., broccoli, turnip, carrot, and tomato) are consumed after thermal processing, which can affect the bioaccessibility and bioavailability of bioactive compounds and, thus, the nutritional value and health benefits of fruit and vegetables. Although some studies have reported a loss of phenols, carotenoids, GLs, and ascorbic acid following thermal processing, others have observed an increase and demonstrated an improvement of the intestinal absorption of nutraceuticals. If the effects of thermal processes on bioaccessibility and bioavailability of bioactive compounds are still unclear, their potential impact on the health effects of fruit and vegetables is even more uncertain and, although widely hypothesized, is far from proven. There are very few observational studies focusing on this topic, and the preclinical evidence is insufficient to suggest a different role of different thermal processes in maintaining the health benefits of fruit and vegetables. Therefore, future comparative studies are needed to fill this gap and identify novel thermal processes that can preserve the amount and structure of metabolites, promote the intestinal absorption of nutraceuticals, and ultimately improve their effects on human health.

## AUTHOR CONTRIBUTIONS

**Federica Narra**: Conceptualization; data curation; visualization; writing—original draft. **Eugenia Piragine**: Conceptualization; data curation; visualization; writing—original draft; investigation. **Giada Benedetti**: Writing—original draft.**Marta Florio** and **Jacopo Spezzini**: Writing—original draft. **Costanza Ceccanti**: Conceptualization; data curation; investigation; visualization; writing—original draft. **Fabiola Troisi**: Writing—original draft; conceptualization; data curation. **Roberto Giovannoni** and **Alma Martelli**: Funding acquisition; writing—review and editing. **Lucia Guidi**: Funding acquisition; writing—review and editing; project administration.

## CONFLICT OF INTEREST STATEMENT

The authors declare no conflicts of interest.
